# Collection and characterization of grapevine genetic resources (*Vitis vinifera*) in the Holy Land, towards the renewal of ancient winemaking practices

**DOI:** 10.1038/srep44463

**Published:** 2017-03-17

**Authors:** Elyashiv Drori, Oshrit Rahimi, Annarita Marrano, Yakov Henig, Hodaya Brauner, Mali Salmon-Divon, Yishay Netzer, Maria Lucia Prazzoli, Maria Stanevsky, Osvaldo Failla, Ehud Weiss, Maria Stella Grando

**Affiliations:** 1 Department of Chemical Engineering and Biotechnology, Ariel University, Ariel, Israel; 2 Agriculture and Oenology Research Dept., Eastern R&D center, Ariel, Israel; 3 Research and Innovation Centre, Fondazione Edmund Mach, San Michele all’Adige, (TN) Italy; 4 Department of Molecular Biology, Ariel university, Ariel, Israel; 5 Department of Agricultural and Environmental Sciences, University of Milan, Italy; 6 The Martin (Szusz) Department of Land of Israel Studies and Archaeology, Bar-Ilan University, Ramat-Gan, Israel; 7 Center Agriculture Food Environment, University of Trento, San Michele all’Adige (TN) Italy

## Abstract

The importance and extent of wine consumption in all life aspects at the Holy Land is well documented. The Muslim influence in this region led to the abandonment of winemaking practices, and possible loss of indigenous wine varieties. Here we present a country wide collection of the local grapevine population including wild and cultivated forms, and its characterization by genetic, ampelographic and enological methods. The ampelographic analysis shows clear differences between Sativa and Sylvestris groups in flower, leaf and cluster parameters, and that most Sativa belong to *proles orientalis*. Genetic population analysis was conducted by analyzing 22 common SSR markers, determining first the unique genotypes, and internally assessing the population’s structure, showing the existence of two distinct Sativa and Sylvestris populations, and a third mixed one. Likewise, the relationship between the Israeli grapevine population and grapevine populations in Europe and parts of Asia was investigated, showing that the Israeli Sativa and Sylvestris populations cluster closely together, suggesting a common genetic source. Lastly, the enological characteristics of selected Sativa and Sylvestris genotypes are presented, demonstrating their potential for quality wine production. This research significantly contributes toward the re-establishment of indigenous and traditional local grapevine varieties into the modern international wine industry.

The domesticated grapevine (*Vitis vinifera* ssp*. sativa*) is one of the classic fruits of the Old World, providing fresh berries, dried raisins, and juice for wine fermentation^1^. Some of the earliest identified locations of domesticated *V. vinifera* come from Early Bronze Age sites in Israel, such as Jericho^2^, Arad^3^ and Lachish^4^. Pre-Bronze Age archaeological remains of grapevine have also been discovered in dozens of sites in Israel and its surrounding area^1^. In addition, a large body of historical evidence for wide viticulture practice can be found in Jewish religious texts, including types of wines which were produced and their exportation destinations^5^,^6^. This testifies to the extent and importance of wine in the diet, religion and culture of the Holy Land. Archaeological evidence for ancient Levantine use of grapes in wine making is also abundant as myriads of ancient winepresses have been found as early as the Bronze Age^7^. Despite hundreds of mentions of winemaking in the Holy Land in ancient texts, there are no clear records as to the varieties used. Starting at the 7^th^ century C.E., Muslim occupation of the region initiated a long period of gradual suppression of wine consumption and production, which is believed to have accelerated with the Mamluk occupation in the 13^th^ century enforcing its strict religious law. This might have led to eventual abandonment of wine grape varieties by local farmers, while maintaining the practice of table grape viticulture^8^. Due to the lack of high quality wine grape varieties in Israel, and for purposes of international marketing, the renewal of the Israeli wine industry by the Baron E. de Rothschild at the end of the 19^th^ century was based solely on international cultivars.

Nevertheless, domestic grape varieties were still grown by the local population for table grapes, grape juice and inexpensive wine production. In the first half of the 20^th^ century, two ampelographic descriptions of local traditional varieties, grown in Israel, Syria and Jordan were published in detail^9^,^10^. Some of these varieties are still cultivated today for the production of table grapes and are preserved in traditional grapevine collections. The Sataf germplasm collection was genetically analyzed in 2008 by Klein and colleagues^11^, giving a first description as to the relatedness of 20 local genotypes to European *V. vinifera* groups. In addition, identification and genetic relationships among several accessions grown for local table grape consumption in the region was also recently reported^12^.

In 2011, we began to conduct an unprecedented nationwide survey to collect and characterize Israeli grapevine germplasm^13^. Our hypothesis in initiating this project was that grape varieties suitable for wine production, neglected during the Muslim prohibition of winemaking, might have survived as table grapes or survived in the wild due to the high resilience of the grapevine plant. Our goal was set to finding these lost grape varieties, genetically verifying that they are unique and indigenous, and choosing the ones suitable for wine production towards renewal of this ancient practice. In our survey, genotypes of both *V. vinifera* ssp. *sativa* (hereafter Sativa) and *V. vinifera* spp. *sylvestris* (hereafter Sylvestris) were collected. Indeed, Sylvestris is considered to be the putative ancestor of the cultivated form and represents the only endemic taxon of the *Vitaceae* in Europe and the Maghreb^14^. During the survey, leaf samples were collected from every vine found for DNA extraction and genetically analyzed using 22 simple sequence repeats (SSRs) loci, commonly used for identification of accessions and population genetic analysis of grapevines^15^. In addition, we collected propagation materials for planting a new germplasm collection at Ariel University. Here we present the ampelographic characterization of several accessions, a genetic population analysis of the Israeli population of grapevines, as well as insights to its relationship with other grape populations found in Europe and parts of Asia. In addition, enological characteristics of some selected Sativa and Sylvestris grapes are shown, representing the potential for quality red and white wine production. These efforts are the first steps toward the re-establishment of the use of indigenous and traditional Israeli grapevine varieties in the modern wine industry.

## Results

### Collection of the germplasm

**Figure 1:**
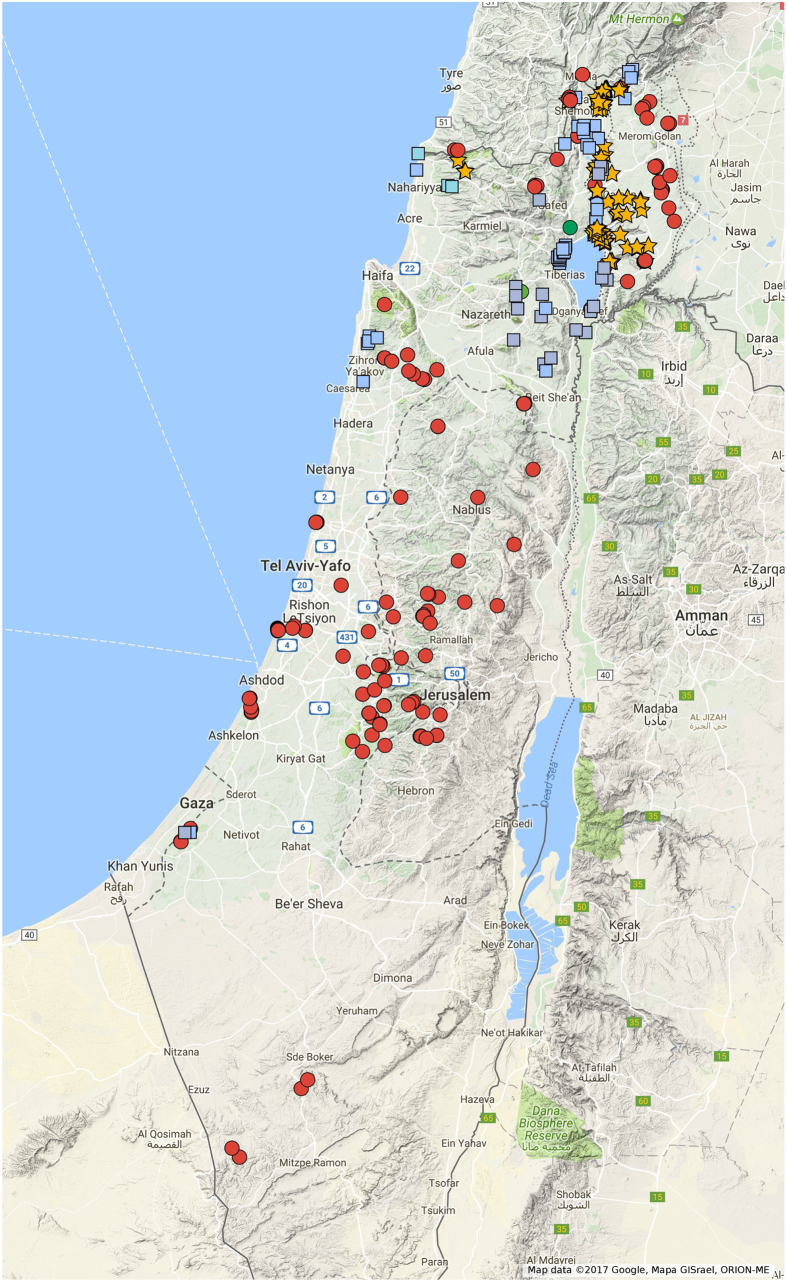
Map of nationwide grape collection. Red dots- *Vitis vinifera ssp. sativa*, Yellow stars-*Vitis vinifera* ssp*. sylvestris*. Blue squares- sampled areas without vinifera findings. Green circles- rootstock findings. The map was created by Google Maps App.

A total of 372 grape accessions (*Vitis* spp.) which were collected in Israel during the years 2011–2015 were analyzed in this work. The collected material consisted of 279 Sativa, including 34 known autochthonous traditional varieties, some of which were previously described^10^. Among accessions, we present here the analysis of 93 putative wild individuals of Sylvestris which is a part of a much larger Sylvestris population collected for future analysis. The Sylvestris accessions were all found in the north of the country, mostly around water sources of the Kineret Lake and the Jordan riverbed (Fig. 1). No Sylvestris accessions were found south of the Samach creek estuary to the Kineret Lake (Fig. 1-blue squares on the map), setting for the first time the southern border of Sylvestris distribution in Israel, and as far as we know the most southern Sylvestris populations in the world. Sativa accessions were collected from the north to south of Israel. The survey resulted in finding Sativa individuals growing in the southern Negev desert in extreme dry conditions, as well as on the southern coastal area in saline conditions (Fig. 1).

### Identification of unique accessions

All accessions were first characterized at 10 SSR loci (VVMD27, VVMD28, VVS2, VVMD7, VMC1B11 VVMD32, VVMD5, VrZAG62, VVMD25, and VrZAG79) in order to identify redundant genotypes. A total of 126 different unique genetic profiles were found, of which 61 were Sativa and 65 were within the Sylvestris population. The genotypes of Sativa included 42 new feral Sativa unknown to local growers. Out of the 34 known autochthonous traditional varieties analyzed, 19 unique genotypes were found, setting 15 varieties as synonyms. The last mentioned cultivars are all preserved in the Sataf and Neve-Yaar collections, as previously described by our group^13^. The comparison of SSR profiles with those reported in the European Vitis Database (www.eu-vitis.de) and in literature revealed that only 11 out of 61 Sativa genotypes were previously described^11^,^12^ (identities are marked in the table of genetic profiles included in the Supplementary data, Table S1.)

### Ampelographic analysis

A collection vineyard containing all unique genotypes was planted in the city of Ariel and will enable us to fully describe all accessions in a few years. In the meantime, a morphological analysis of 55 accessions in their site of discovery was conducted using 17 OIV descriptors that coded according to COST Action GrapeNet FA1003 (2010–14)^16^. These accessions were chosen due to their relative availability and ease of accessibility for descriptive analysis in the wild. Considering the classification scheme of domestic grapevines proposed by Negrul^17^ and mainly bearing in mind the berry and bunch traits, 23 Sativa accessions were classified as follows: 16 in the *proles orientalis*, 5 in the *proles pontica* and 2 in *proles occidentalis* (Table S2).

**Figure 2:**
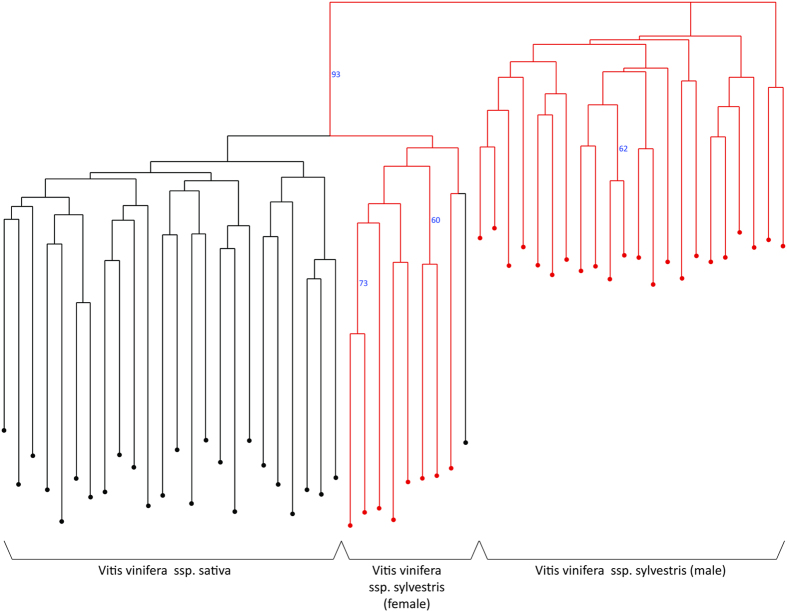
Phenotype analysis. Neighbor-joining dendrogram based on simple matching dissimilarity matrix calculated from the dataset of 17 OIV descriptors across 55 unique accessions. Black - *Vitis vinifera ssp. sativa*, red - *Vitis vinifera ssp. sylvestris*.

**Figure 3:**
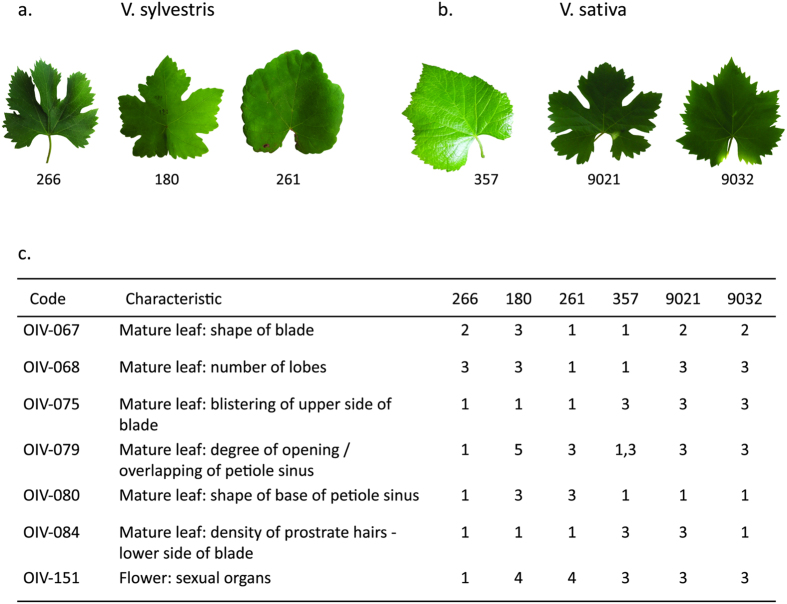
High polymorphism in leaves among Sylvestris and Sativa accessions. (**a**) Leaves of *Vitis vinifera ssp. sylvestris*., (**b**) Leaves of *Vitis vinifera ssp. sativa*., (**c**). Summary table of the ampelographic mean values of OIV codes for leaves presented above.

**Figure 4:**
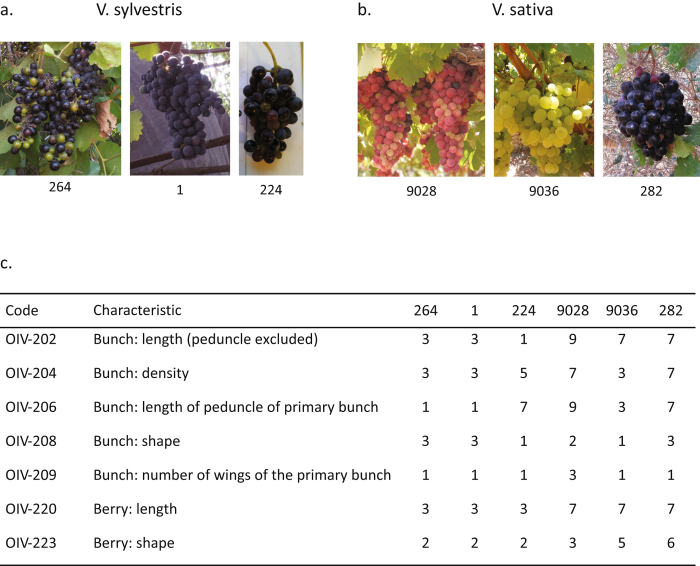
High polymorphism in bunches among Sylvestris and Sativa accessions. (**a**) Bunches of *Vitis vinifera ssp. sylvestris*., (**b**) Bunches of *Vitis vinifera ssp. sativa*., (**c**). Summary table of the ampelographic mean values of OIV codes for bunches presented above.

Using the OIV descriptors data, a neighbor-joining dendrogram based on simple matching dissimilarity matrix was calculated (Fig. 2). The dendrogram divides the population into 3 main groups; the Sativa population is separated from the Sylvestris groups, which are classically divided into male and female plants. Considering the morphological traits measured for the different populations, a few criteria were much effective in distinguishing the Sativa from the Sylvestris accessions: a. Sativa grapevines have perfect synecious flowers, whereas Sylvestris are dioecious plants, b. Sativa accessions have higher amount of blistering of upper side of leaf blade (Fig. 3) and c. all Sylvestris accessions have short clusters with short, round berries, whereas most Sativa accessions have long clusters and long ovule berries (Fig. 4). Interestingly, Sylvestris accession bunches differ in shape and structure; whereas some show characteristic thin clusters with scattered berries, others boast thicker bunches with dense berry array (Fig. 4a). Leaf shapes and size vary greatly amongst the Sylvestris forms, while more uniformity is found amongst the Sativa (Fig. 3).

### Population genetic analysis

**Figure 5:**
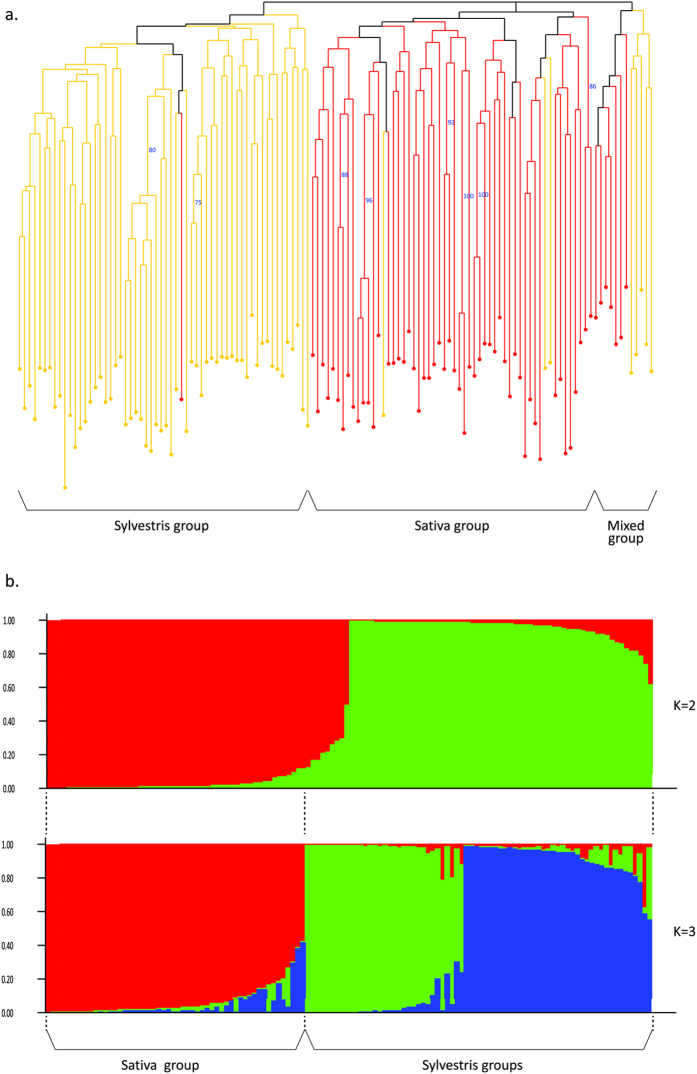
Population analysis of Israeli germplasm collection. (**a**) Neighbor-joining dendrogram based on simple matching dissimilarity matrix calculated from the dataset of 22 SSR across 126 genotypes. Red dots- *Vitis vinifera ssp. sativa.* Yellow dots-*Vitis vinifera* ssp*. sylvestris.* (**b**) Population structure of the collection using the model-based program structure.

To further characterize the final panel of 126 unique genotypes, 12 additional SSR loci were genotyped. We used the data derived from the full set of commonly used 22 SSR loci^15^ to estimate the main indexes of genetic diversity in cultivated and wild grapevine groups and to assess how they are related to each other. Sativa and Sylvestris populations revealed almost the same values of expected and observed heterozygosity, while the fixation index of the Sylvestris population was slightly higher than that of the Sativa population (0.07, 0.06, respectively) (Table 1). Genetic profiles were used for Bayesian clustering analysis implemented in STRUCTURE. The most likely number of clusters (K), obtained using the ΔK method proposed by Evanno *et al*.^18^, were equal to K = 2 and K = 3 (Fig. 5b).

A neighbor-joining unweighted tree was built based on the SSR profiles (Fig. 5a). A general differentiation was observed between Sativa (red), Sylvestris (yellow) and mixed subpopulations. A further subdivision within the two main groups was highlighted: the Sativa accessions were split into two distinct main subgroups, while the Sylvestris profiles were distributed across three cascading subgroups. One Sativa accession (389) fell within the Sylvestris main clade, while some Sylvestris accessions (141 and 142 in the upper subgroup and 180 in the second subgroup) were found within the main Sativa group. Five Sylvestris (192,189, 276, 3, 266), and 7 Sativa individuals (220, 9034, 46, 9036, 9019, 5, 15) clustered in a third separated group.

### Comparison to neighboring Vitis populations

**Figure 6:**
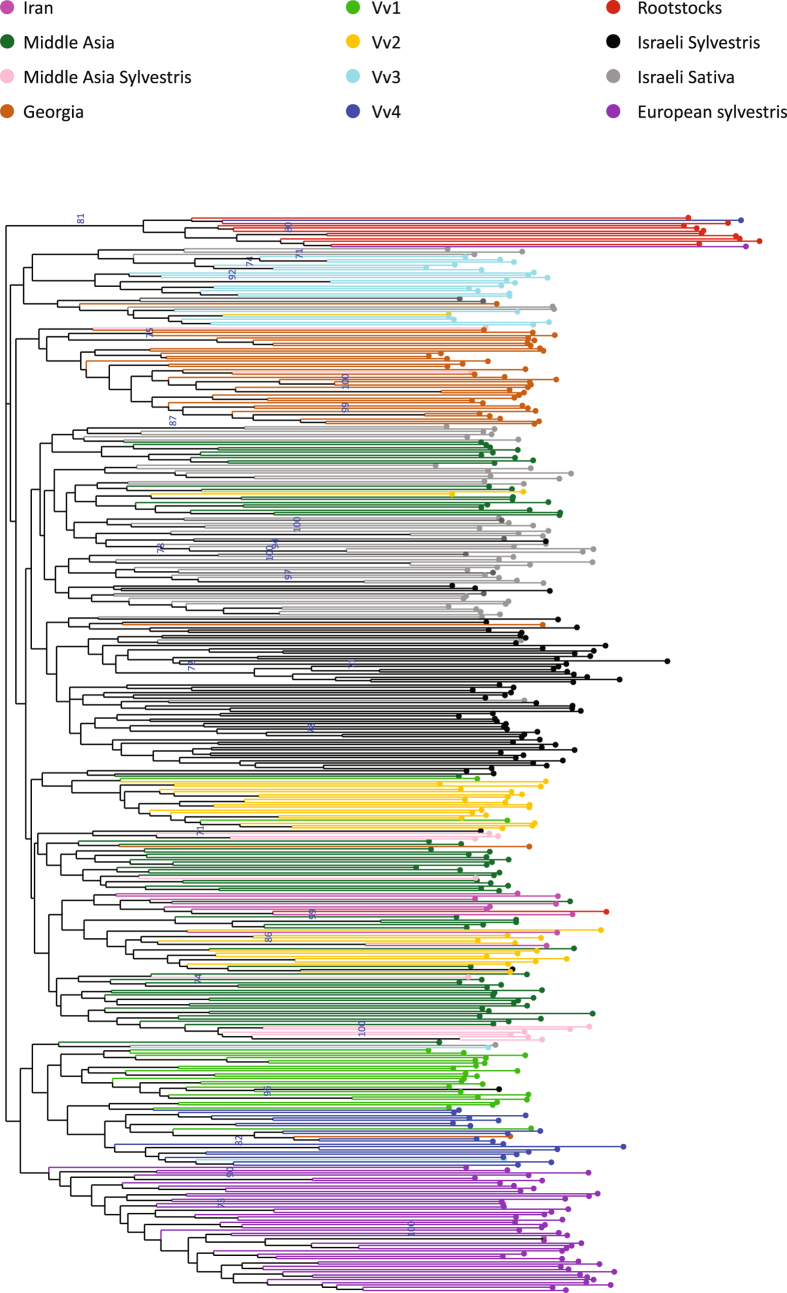
A wide regional genetic population analysis. Neighbor-joining dendrogram based on simple matching dissimilarity matrix calculated from the dataset of 22 SSR across 411 genotypes was conducted. The Israeli grape germplasm collection was analyzed alongside grape germplasm collections of FEM (Vv1-4, European Sylvestris), Iran, Georgia and Middle Asia (Sativa and Sylvestris). A rootstock population was used as an outgroup.

To gain a broader understanding of the genetic relationship between the Israeli *Vitis* germplasm and other important *Vitis* populations in Eurasia, we performed an additional cluster analysis including homologous SSR profiles of 278 Eurasian grapevines (Fig. 6). These accessions belong to four ancestral subpopulations of Sativa (Vv) and one subset of Sylvestris (almost all from the Italian Peninsula), detected within a large sample of grapevine accessions through a hierarchical clustering approach^15^. In particular, we included a cluster of Italian and Greek wine grapes (Vv1), representing the proles *pontica*, French and German wine varieties (Vv4), representing the proles *occidentalis*, and table and wine Muscat grapes (Vv3) reflecting the proles *orientalis* sub pr. *caspica*. Subpopulation Vv2 was composed of both table grape varieties (proles *orientalis* sub pr. *antasiatica*) and Spanish wine grapes. In addition, we included 10 diverse cultivars from the Sistan area of Iran and a set of 119 wild and cultivated genotypes originating from Georgia and Middle Asia (MA), already investigated by Marrano *et al*.^19^. In this broader context (Fig. 6), the Israeli Sylvestris and Sativa populations clustered closely as two distinct groups. Furthermore, 4 Israeli Sylvestris, 19 Middle Asia and 2 Vv2 accessions fell within the main Israeli Sativa group, while 2 Israeli Sativa and one Georgian accession clustered within the main Israeli Sylvestris group. Moreover, the Israeli Sativa and Sylvestris groups clustered closely with a large group including the MA Sativa and Sylvestris accessions, the Iranian subset and most of the Vv2 accessions, while 7 Israeli Sativa accessions fell within the Vv3 population cluster. On the other hand, the Georgian subpopulation formed a separated group. The Italian Sylvestris group stands alone and close to a subgroup including the main bodies of both Vv1 and Vv4 populations.

### Identification of varieties with winemaking potential

Following the determination of unique populations, vines containing a sufficient amount of grapes were harvested for micro-vinification. Wine characteristics were analyzed to help in determining the potential of quality wine making of each variety. Here we show the analysis of some red and white varieties, micro-vinificated during 2014 harvest (Table 2). In the Sativa group, varieties Sorek, Misla and Yael show potential for quality red winemaking, as their basic parameters of alcohol and acid are in normal range, and they contain high levels of color and total polyphenols, which are important parameters for wine quality and aging potential. Most white Sativa varieties show good basic wine potential characteristics, producing wines with appropriated amounts of alcohol and acid. In this group, we included the Jandali and Hamdani traditional varieties, used today as table grapes by Arab population.

## Discussion

Our broad survey of *Vitis vinifera* in Israel generated a large population of diverse genotypes. The Sativa population consists of two sub groups, the first of which is composed by traditional Sativa varieties, mainly considered as table grapes. Among these, there are historical indications of use of the Hamdani and Jandali varieties for winemaking in Jerusalem during the 2^nd^ century C.E.^20^. These varieties can be still used to produce good quality wines with modern technologies, as suggested by our analysis. The second subgroup consists of feral Sativa, unknown to local growers (thus having no names yet), found during our survey in the wilds. We consider these accessions to consist both of escaped ancient cultivars, as some accessions with identical genotypes were found in distant locations, and possibly some natural crosses.

The investigated grapevine collection included also Sylvestris accessions, the distribution of which is restricted to the Northern part of Israel. The reason for the restriction of the Sylvestris population to the above described area is unclear. Biagini *et al*.^21^ found that, in accordance with previous studies^22^,^23^, Sylvestris grows wild in moist habitats and close to major rivers. Apparently the soil type is also a crucial factor, as in other works nearly half of the Sylvestris were found growing in alluvial soils^21^. In addition, it is well known that cultivated grapevines show differential resistance and physiological response in saline soils^24^,^25^,^26^. We believe that environmental abiotic factors, primarily water and soil availability and salinity, may play a crucial role in the restricted distribution pattern of Sylvestris in Israel.

Ampelographic analysis showed distinct differences between Sativa and Sylvestris populations, mostly in flower, leaf and cluster properties. These distinct differences between the Sativa and Sylvestris populations, shown by the Neighbor-joining dendrogram assure us of further classification of each individual to its correct grouping during genetic analysis.

Classification of the accessions to *proleses* was conducted following Negrul’s taxonomical system which has been independently validated by several other authors^27^,^28^,^29^,^30^. The most relevant discriminant phenotypes considered in the present survey are related to the berry and the bunch. Most Israeli accessions were found to belong to *proles orientalis*. Indeed, with accordance to our phenotypic observations, the wide genetic population analysis shows that 7 of the Israeli Sativa varieties cluster within the Vv3 group, which reflects *proles orientalis subproles caspica*. Some other few accessions were found to belong to the *proles pontica* and *proles occidentalis*. This polymorphism found in the Israeli population of Sativa can suggest that the initial *orientalis*-based population gained representatives of other *proleses*, as discussed below.

The neighbor-joining genetic analysis of the Israeli population based on SSR markers shows a division to a main Sativa group, a main Sylvestris and a third of mixed origin. We cannot find a clear geographical explanation to the existence of the mixed group. Indeed, 3 of the 5 Sylvestris in this group come from the Banias creek in the north, were we found both Sativa and Sylvestris growing alongside each other, but the other two Sylvestris come from the northern part of Lake Kinert, were no Sativa exist, and the Sativa accessions in this mixed group are mostly from southern regions where no Sylvestris do exist. Thus, it is not likely that the genetic relatedness is caused by natural crossings of Sylvestris with local Sativa individuals, and another explanation suggesting a common genetic source might be in favor. The above-mentioned division is also apparent from the analysis of the most likely number of clusters (K), which also indicates the existence of a Sylvestris group including 11 Sativa.

It is worth noting that these Sativa and Sylvestris populations, beside a similar high level of genetic diversity as quantified by the expected heterozygosity, also have similar average of observed heterozygosity. These are positive indicators for the status of the wild group, as reduction in observed heterozygosity has been reported in wild grapevines analyzed in Spain, Portugal, France and Italy^31^, most likely due to the reduction of the wild populations by human action.

The genetic analysis of the Israeli collection against the European and Asian datasets shows that the Israeli Sylvestris and Sativa populations are genetically proximal. This evidence, as well as the relatedness found above in the internal genetic analysis, might suggest a local domestication in the Holy Land. This claim is supportive of the multi-local selection and domestication theory as previously proposed for the Iberian Peninsula^27^,^32^. Our hypothesis can be further supported by the fact that opposing to European Sylvestris, which are generally considered unsuitable for wine production due to low sugar levels and high acidity^33^,^34^, many Sylvestris accessions in Israel are naturally suitable for wine production, as was demonstrated with wines made from Sylvestris accessions, presented in our work. The readiness of the Sylvestris grapes in Israel for consumption and wine making makes them natural candidates for harvest, regular use and later domestication. Indeed, many prehistoric archaeological findings of Sylvestris grapes were found in Israel, as well as Sativa findings starting from the early Bronze Age, as mentioned above.

In addition, the genetic analysis showed that the main Israeli Sativa cluster also includes several accessions from Middle Asia, as also noted in the *proles* characterizations above. This may suggest a flow of varieties originating in the Holy Land – a bridge between Africa, Asia and Europe which was conquered intensively by various empires. Indeed, the combined Israeli collection clustered within a large group including the other Vv2 genotypes, MA Sativa and Sylvestris populations and the Iranian varieties. Proximity and easy land access from these regions to the Levant may have favored the spread of plant material in both directions.

Lastly, grapes fermented from some of the Sativa varieties collected in their original site show winemaking potential. The color and polyphenol characteristics of Ein-Misla, Sorek and Nitzan unique native varieties are similar or better than those of a local Cabernet Sauvignon wine used as reference. This initial potential would be further pursued and verified in the near future towards integration of these varieties into the wine industry. As mentioned above, the wines produced from some Sylvestris accessions gave rise to wines with normal alcohol and acid levels, and high polyphenolic content. After due selection, this can lead to the integration of Israeli Sylvestris cultivars into the general wine industry, bringing with them possible stress resistance, quality and health traits.

## Materials and Methods

### Plant material

A total of 372 accessions of grapevine (*Vitis* spp.) were collected across Israel during the years 2011–2015. For DNA extraction, shoot tips were excised from each accession on site, usually during the spring, and kept on ice until storage at −80 °C was available.

### Mapping and phenotyping

Accessions were localized on a map using the free application Google Maps (Google, Inc.). Accessions were characterized morphologically according to the OIV standards adopted by the “COST Action GrapeNet FA1003” (2010–2014)^16^. The examined criteria were the following: OIV4, OIV51, OIV53, OIV67, OIV68, OIV75, OIV79, OIV80, OIV84, OIV151, OIV202, OIV204, OIV206, OIV208, OIV209, OIV220 and OIV223. The OIV descriptors refer to shoot tips, young shoots, and young and mature leaves. In several accessions bunches, berries and wine parameters were checked.

### DNA extraction protocol

Frozen leaves (70 mg) were maintained at −80 °C and weighed in 1.5 ml microtubes. The tissue was then ground with a pestle diluted with 700 μl of CTAB buffer (0.05 M Tris-HCl pH 8.0, 1.1 M NaCl, 0.05 M ethylenediaminetetraacetic acid (EDTA), 0.4 M LiCl, 1% polyvinylpyrrolidone (PVP), cetyltrimethyl ammonium bromide (CTAB), 1% β-mercaptoethanol)^11^. Tubes were incubated at 65 °C for 30 min and periodically vortexed. An equal volume of chlorophorm:isoamyl alcohol (24:1) was added, samples were centrifuged at 10000X g for 13 min. NaCl (0.5 volumes of 5 M) and 100% cold ethanol (2 volumes; −20 °C) were added to the aqueous phase, samples were mixed and incubated at −20 °C for 30 min., centrifuged and supernatant was discarded. DNA pellets were washed twice with 70% cold ethanol (−20 °C), resulting DNA pellets were air-dried at room temperature and dissolved in 70 μl of DNase free water (Promega).

### SSR genotyping

Twenty two SSR markers previously applied for grape germplasm characterization were chosen^15^. Nine multiplex panels of fluorescent-labeled microsatellite loci were used. Two controls (Merlot, Cabernet Sauvignon) were added to each multiplex run in order to harmonize SSR allele sizes and compare molecular genotypes among different studies and databases. Multiplex PCR amplifications were performed in a final volume of 25 μl containing 50–100 ng of genomic DNA, 12.5 μM Go Taq Green Master Mix (Promega), and 0.4 μM of each primer. Reactions were performed on a T100™ Thermal Cycler (Bio-Rad) using the following program: a hot start of 95 °C for 7 min, 30 amplification cycles of 45 sec at 95 °C, 1 min at 56 °C, 30 sec at 72 °C, and a final extension step of 30 min at 72 °C. PCR products were diluted 1:5 in sterile water and 1 μl of these dilutions were mixed with 9.5 μl of formamide and 0.3 μl of the GeneScan™ 500 ROX^W^ Size Standard (Applied Biosystems). DNA fragments were denatured and size-fractionated using capillary electrophoresis on an ABI 3500 Genetic Analyzer (Applied Biosystems). GeneMapper 5 (Applied Biosystems) was then used for allele size estimation and chromatograms were read by two independent raters. Determinations of unique accessions were performed using the “Excel Microsatellite Toolkit”^34^,^35^. Rates of missing data (MD) were below 4%. Identification of the collected varieties was based on the pairwise comparison with SSR profiles in the European Vitis Database (www.eu-vitis.de).

### Analysis of genetic diversity and population structure

The final dataset of non-redundant genotypes at 22 SSR loci was used to estimate the main diversity statistics, as well as most likely subdivision (K) by using STRUCTURE 2.1 software^36^. STRUCTURE sorts individuals into K clusters, according to their genetic similarity. Ten independent runs for K values ranging from 1 to 20 were performed with a burn-in length of 50000 followed by 500000 iterations. The true K was calculated based on the second order rate of change of the likelihood (ΔK), method proposed by Evanno *et al*.^18^. A weighted neighbor-joining tree was constructed based on the simple-matching dissimilarity matrix with 10000 bootstrap replicates. Further cluster analysis was performed and included the genetic profiles of 112cultivars and 47 wild individuals which belong to the FEM germplasm collection (ITA362)^15^, 40 Georgian varieties, and 79 grapevine genotypes from Central Asia^37^ (see Table S3). In addition, the SSR profiles of 11 grape rootstocks (Vitis spp.) were used for out-group comparisons.

### Vinification

As grapes were harvested from wild or feral vines, the amount of grapes processed for winemaking was between 5–50 kg, depending on the vines prosperity.

Whole bunches were harvested at appropriate sugar levels for winemaking, destemmed and crushed. White grapes were immediately pressed by a hydraulic press and juice cooled to 8 °C, incubated overnight, then decanted and kept at 15 °C for the fermentation period. 20 gr of the commercial *Saccharomyces cerevisiae* strain BDX (Lallemand, Montreal, Canada) per 100 kg grapes was used for vinification of red varieties, and Lalvine QA23 (Lallemand, Montreal, Canada) in the same dosage was used for whites. The cap punching operations were carried out three times a day during maceration of red varieties, which was conducted in a temperature controlled room at 25 °C. Eight days after commencing alcoholic fermentation of red varieties, wine was obtained by pressing the must using a hydraulic press machine, and kept at the same temperature until density dropped under 0.994. Immediately after press for the whites, and following completion of malolactic fermentation for the reds, sulfur dioxide was added to the wine at 60 mg/l and the wine was syphoned a week later and stored at 15 °C until used for further analyses.

### Wine analysis

Ethanol in wine samples was determined using a Super Dee digital distillator and Super Alcomat electronic hydrostatic balance (Gibertini, Italy). Titratable acidity (TA) and pH were measured using a Hana HI 2211 pH meter (Hanna Instruments, USA). TA is expressed as tartaric acid in g/l and was determined by diluting the 10 ml of wine with 10 ml of distilled water and by subsequent titration with 0.1 M NaOH to pH 8.2. Color characteristics of wines were obtained by spectrophotometer (GENESYS™ 10 S UV-Vis Spectrophotometer, Thermo Scientific). Color density was calculated as total of absorbance of red and yellow pigments (A_520nm_ + A_420nm_). Color hue was defined as the ratio intensities of yellow to red pigments (A_420nm_/A_520nm_)^38^. To determine total phenolics (mg/l) in the wines, samples were analyzed at 280 nm and concentrations calculated from a calibration curve constructed using dilutions of Gallic acid in 10% ethanol^39^.

**Accession codes.** (accession codes for all plant material are included in supplementary data table S1).

## Additional Information

**How to cite this article:** Drori, E. *et al*. Collection and characterization of grapevine genetic resources (*Vitis vinifera*) in the Holy Land, towards the renewal of ancient winemaking practices. *Sci. Rep.* **7**, 44463; doi: 10.1038/srep44463 (2017).

**Publisher's note:** Springer Nature remains neutral with regard to jurisdictional claims in published maps and institutional affiliations.

## Supplementary Material

Supplementary Tables

## Figures and Tables

**Table 1:** Summary statistics of genetic variation at 22 SSR loci in the Israeli germplasm collection.

Sample	N	Na	Ne	Ho	He	F
Sativa	61	11.41	5.40	0.73	0.77	0.06
Sylvestris	65	11.05	5.47	0.72	0.78	0.07
Total	126					

N – sample size; Na – mean number of alleles per locus; Ne - number of effective alleles; Ho – observed heterozygosity; He – unbiased expected heterozygosity; F – fixation index (inbreeding coefficient).

**Table 2:** Wine characteristics of some selected Israeli varieties.

		Accession number/Name	Ethanol, %	pH	TA g/l	Color density, AU	Color hue	Total phenols, mg/l
**Sativa**	**Red wines**	Yael- 19	11.0	3.62	3.8	7.2	0.6	729.5
		El-al- 282	11.1	3.52	6.0	2.5	1.0	573.0
		Misla- 138	11.9	3.52	4.5	8.2	0.7	891.0
		Sorek- 118	11.5	3.88	2.6	8.6	0.7	1101.1
		**Bituni**	11.5	4.20	2.3	1.8	0.8	511.1
	**White wines**	**Jandali**	11.7	3.31	4.9			
		**Hamdani**	11.4	3.30	5.3			
		Nitzanim -286	13.5	3.44	4.9			
		Bustan- 16	12.0	3.46	3.9			
		Misla W-116	12.9	3.47	4.1			
**Sylvestris**	**Red wines**	Pomela- 1	12.1	3.60	5.9	10.8	0.9	2062.2
		Michnaf -2	14.4	3.72	6.8	15.3	0.9	2266.2
		C.T. knisa	14.1	3.73	6.3	21.7	1.2	1819.1
		C.T. kane	11.2	3.14	7.3	10.9	0.6	1724.1
		Cabernet S. (Shilo)	13.6	3.73	5.5	5.0	0.8	927.8

Wines were produced by micro-vinification methods using grapes harvested from vines growing naturally in the wild. Wines were analyzed 6 months after production. Cabernet Sauvignon wine, used as a reference, was made from grapes harvested in Shilo, Israel. In bold - traditional indigenous varieties.

## References

[b1] Zohary D., Hopf M. & Weiss E. Domestication of Plants in the Old World. (Oxford University Press, 2012).

[b2] Hopf M. In Excavations at Jericho(eds. Knyon, & Holland, ) 576–621 (1983).

[b3] Hopf M. In Erly Arad I. The chalcolithic settlement and early Bronze Age city.(ed. Amiran, ) 64–82 (Israel Exploration Society, 1978).

[b4] Helbaek H. In Lachish (Tell ed-Duweir) IV: the Bronze Age.(ed. Turfnell, ) 309–317 (1958).

[b5] Heleck W. Die Beziehungen Ägyptens zu Vorderasien i, 3. und 2. Jahrtausend v. Chr. Weisbaden (1971).

[b6] Ahituv S. Economic Factors in the Egyptian Conquest of Canaan. Israel Exploration Journal 28, 93–105 (1978).

[b7] Frankel R. Presses for Oil and Wine in the Southern Levant in the Byzantine Period. Dumbarton Oaks Papers 51, 73–84 (1997).

[b8] Amar Z. In The agricultural produce in the land of Israel in the middle ages. 100–135 (Yad Izhak Ben-Zvi, [Hebrew] 2000).

[b9] Weitz Y. Hagefen.(Mitzpe & Bustenai, [Hebrew] 1931).

[b10] Hochberg N. Growing of the grapevine.(Hasade, [Hebrew] 1954).

[b11] Klein B. Y., Ben-Yair C., Bar-Gal G. K. & Greenblatt C. L. Microsatellite genotyping of cultivars of the Holy Land grapevine, *Vitis vinifera* ssp. sativa (Vitaceae). Botanical Journal of the Linnean Society 156, 513–521 (2008).

[b12] Basheer-Salimia R. . Molecular identification and genetic relationships of Palestinian grapevine cultivars. Molecular biotechnology 56, 546–56 (2014). 24469973 10.1007/s12033-013-9728-7

[b13] Drori E. . Ampelographic and genetic characterization of the Israeli grapevine germplasm collection. Vitis(2015).

[b14] Heywood V. H. & Zohary D. A catalogue of the wild relatives of cultivated plants native to Europe. Flora Medit 5, 375–415 (1995).

[b15] Emanuelli F. . Genetic diversity and population structure assessed by SSR and SNP markers in a large germplasm collection of grape. BMC plant biology 13, 39 (2013). 23497049 10.1186/1471-2229-13-39 PMC3610244

[b16] COST Action FA1003 GRAPENET: East-West Collaboration for Grapevine Diversity Exploration and Mobilization of Adaptive Traits for Breeding (2010–2014).

[b17] Negrul A. M. In Ampelography of the Soviet Union(eds. Baranov, , Kai, , Lazarevski, , Palibin, & Prosmoserdov, ) (Pischepromizdat, 1946).

[b18] Evanno G., Regnaut S. & Goudet J. Detecting the number of clusters of individuals using the software structure: a simulation study. Mol Ecol 14 (2005). 10.1111/j.1365-294X.2005.02553.x 15969739

[b19] Marrano A., Grzeskowiak L., Sanz P., Lorenzi S., Prazzoli M. . Genetic diversity and relationships in the grapevine germplasm collection from Central Asia. Vitis - Journal of Grapevine Research 54, 233–237 (2015).

[b20] Amar Z., Drori E., Netzer Y., Henig Y. & Segev A. Jandali and Hamdani - Varieties of Table and Wine Grapes in Past and Present. Judea and Sameria Research Studies 23, 399–408, [Hebrew] (2014).

[b21] Biagini B., De Lorenzis G., Imazio S., Failla O. & Scienza a. Italian wild grapevine (*Vitis vinifera* L. subsp. sylvestris) population: insights into eco-geographical aspects and genetic structure. Tree Genetics & Genomes 1369–1385, doi: 10.1007/s11295-014-0767-4 (2014).

[b22] Anzani R., Failla O., Scienza A. & De Micheli L. Individuazione e conservacione del germoplasma di vite selvatica. Vitis vinifera silvestris 51–60 (1993).

[b23] Arnold C. Ecologie de la vignesauvage, Vitis vinifera L. ssp. sylvestris (Gmelin) Hegi, dans le forêtalluvialestecolluvialesd’Europe. (University of Neuchâtel, 2002).

[b24] Downton W. J. S. & Loveys B. R. Abscisic Acid Content and Osmotic Relations of Salt-Stressed Grapevine Leaves. Functional Plant Biology 8, 443–452 (1981).

[b25] Shani U. & Ben-Gal A. Long-term Response of Grapevines to Salinity: Osmotic Effects and Ion Toxicity. American Journal of Enology and Viticulture 56, 148–154 (2005).

[b26] Askri H. . Short-term response of wild grapevines (*Vitis vinifera* L. ssp. sylvestris) to NaCl salinity exposure: Changes of some physiological and molecular characteristics. Acta Physiologiae Plantarum 34, 957–968 (2012).

[b27] Arroyo-García R. . Multiple origins of cultivated grapevine (*Vitis vinifera* L. ssp. sativa) based on chloroplast DNA polymorphisms. Molecular ecology 15, 3707–14 (2006). 17032268 10.1111/j.1365-294X.2006.03049.x

[b28] Bacilieri R. . Genetic structure in cultivated grapevines is linked to geography and human selection. BMC Plant Biology 13, 1–14 (2013). 23394135 10.1186/1471-2229-13-25 PMC3598926

[b29] Imazio S., Labra M., Grassi F., Scienza A. & Failla O. Chloroplast microsatellites to investigate the origin of grapevine. Genet Resour Crop Evol 53 (2006).

[b30] Myles S. . Genetic structure and domestication history of the grape. Proceedings of the National Academy of Sciences of the United States of America 108, 3530–3535 (2011). 21245334 10.1073/pnas.1009363108 PMC3048109

[b31] Arroyo-García R. a. & Revilla & The Current Status of Wild Grapevine Populations (*Vitis vinifera* ssp sylvestris) in the Mediterranean Basin. The Mediterranean Genetic Code - Grapevine and Olive 51–72, doi: 10.5772/3442 (2013).

[b32] Grassi F. . Evidence of a secondary grapevine domestication centre detected by SSR analysis. Theoretical and Applied Genetics 107, 1315–1320 (2003). 13679993 10.1007/s00122-003-1321-1

[b33] McGovern P. Ancient wine: the search for the origins of viniculture. (Princeton University Press, 2004).

[b34] Ocete R. . Characterization of *Vitis vinifera* L. subspecies sylvestris (Gmelin) Hegi in the Ebro river Basin (Spain). Vitis 50, 11–16 (2011).

[b35] Park S. D. E. The Excel Microsatellite Toolkit (2001).

[b36] Pritchard J. K., Stephens M. & Donnelly P. Inference of population structure using multilocus genotype data. Genetics 155, 945–959 (2000). 10835412 10.1093/genetics/155.2.945 PMC1461096

[b37] Marrano A. . Genetic diversity and relationships in the grapevine germplasm collection from Central Asia. VITIS - Journal of Grapevine Research 54, 233–237 (2015).

[b38] Jacobson J. L. Introduction to wine laboratory practices and procedures. Introduction to Wine Laboratory Practices and Procedures(Springer US, 2006).

[b39] Cliff M. A., King M. C. & Schlosser J. Anthocyanin, phenolic composition, colour measurement and sensory analysis of BC commercial red wines. Food Research International 40, 92–100 (2007).

