# Supplementary material for: Collection and characterization of grapevine genetic resources (Vitis vinifera) in the Holy Land, towards the renewal of ancient winemaking practices
Source: Sci Rep. 2017 Mar 17;7:44463. doi: 10.1038/srep44463 (PMC5358690; doi:10.1038/srep44463)
Supplement: Supplementary Tables [file srep44463-s1.pdf]

Collection and characterization of grapevine genetic resources (*Vitis vinifera*) in the Holy Land, towards the renewal of ancient winemaking practices.

Elyashiv Drori[a,f*], Oshrit Rahimi[f], Annarita Marrano[b], Yakov Henig[f], Hodaya Brauner[f], Mali Salmon-Divon[e], Yishay Netzer[a,f], Maria Lucia Prazzoli[b], Maria Stanevsky[f], Osvaldo Failla[c], Ehud Weiss[d] and Maria Stella Grando[b].

[a] Department of Chemical Engineering and Biotechnology, Ariel University
[b]Research and Innovation Centre, Fondazione Edmund Mach, San Michele all'Adige (TN) Italy
[c]Department of Agricultural and Environmental Sciences, University of Milan, Italy
[d]The Martin (Szusz) Department of Land of Israel Studies and Archaeology, Bar-Ilan University, Ramat-Gan, Israel
[e]Department of Molecular Biology, Ariel university, Ariel, Israel
[f]Agriculture and Oenology Research Dept., Eastern R&D center, Ariel, Israel

[*] Corresponding author: Elyashiv Drori, Eastern R&D center, Ariel, Israel 40700. 972-543231686 shividrori@gmail.com

Table S1. Genetic profiling data set

SATIVA		SSR loci																									
**Accession name / code**	**Internal code**	**VVMD27**	**VVMD27**	**VVMD28**	**VVMD28**	**VVS2**	**VVS2**	**VVMD7**	**VVMD7**	**VVMD32**	**VVMD32**	**VMC1B11**	**VMC1B11**	**VVMD5**	**VVMD5**	**VrZAG62**	**VrZAG62**	**VVMD25**	**VVMD25**	**VrZAG79**	**VrZAG79**	**VVMD24**	**VVMD24**	**VVIN16**	**VVIN16**	**VVIQ52**	**VVIQ52**
BITUNI TORAN / ISR36-2013-9004	**9004**	183	191	245	259	132	156	247	249	261	271	167	183	238	238	185	187	246	256	239	257	211	217	151	151	80	80
HADARI / ISR36-2013-9006	**9006**	177	177	245	245	148	154	247	249	257	271	181	187	228	236	191	203	242	246	245	245	215	221	153	153	78	84
DUMIAT / ISR36-2013-9007	**9007**	177	177	245	259	128	148	239	247	251	263	167	179	246	246	191	195	242	242	239	247	203	207	149	151	80	82
MAATAR / ISR36-2013-9008	**9008**	177	191	237	245	140	150	233	251	251	257	167	181	226	238	201	203	246	256	247	257	211	217	149	153	80	84
SAFADI / ISR36-2013-9009	**9009**	179	191	247	249	150	150	247	251	257	271	171	173	238	238	187	203	250	250	251	257	207	211	149	151	80	84
MARAWANI / ISR36-2013-9011	**9011**	191	191	237	245	132	140	239	249	271	273	171	187	226	236	187	199	240	246	247	257	207	217	153	153	84	84
BALUTI / ISR36-2013-9012	**9012**	177	177	245	259	140	148	233	247	261	261	167	171	236	238	191	203	242	246	245	253	211	215	151	153	72	80
ZEITUNI / ISR36-2013-9013	**9013**	177	191	245	259	130	140	239	243	259	263	167	171	238	246	187	195	246	256	239	249	203	221	149	149	78	82
RUMI / ISR36-2013-9015	**9015**	179	183	259	259	150	152	243	251	255	271	167	189	238	240	187	203	246	256	249	249	207	207	151	151	80	84
MIDWAR / ISR36-2013-9017	**9017**	177	191	259	271	128	152	243	247	263	263	167	167	226	246	187	191	240	246	239	249	211	221	157	157	78	78
TOFACHI / ISR36-2013-9018	**9018**	177	191	245	245	140	150	233	247	251	271	167	171	234	246	195	203	240	250	247	257	207	211	149	153	80	80
DABOUKI MASRIK / ISR36-2013-9019	**9019**	177	181	259	259	132	150	247	249	249	271	167	187	232	234	187	203	242	246	245	245	207	221	151	153	80	82
DARWISHI / ISR36-2013-9024	**9024**	177	177	259	271	128	148	247	247	263	263	167	171	236	236	191	191	240	242	239	255	211	217	157	157	78	82
SIKUSI / ISR36-2013-9026	**9026**	181	181	245	259	132	140	233	247	271	271	171	189	236	246	203	203	240	242	247	257	211	221	149	153	80	82
HILWANI / ISR36-2013-9028	**9028**	191	191	251	259	144	150	239	251	251	257	167	181	232	240	191	203	240	250	251	257	207	207	149	151	78	80
TAMAR CHULATA / ISR36-2013-9029	**9029**	183	183	235	259	130	132	239	249	259	271	185	185	224	232	185	187	250	256	243	251	207	207	151	151	78	80
ZENI ARUV / ISR36-2013-9031	**9031**	191	191	247	259	148	154	245	249	263	271	167	175	222	234	187	191	242	246	251	257	211	211	149	157	82	82
JANDALI / ISR36-2013-9034	**9034**	183	183	259	259	132	150	245	249	251	271	171	187	236	240	199	199	242	242	247	255	207	221	153	153	78	82
MARAWI / ISR36-2013-9036	**9036**	177	183	259	259	140	150	247	249	249	271	167	187	226	234	187	203	246	250	247	247	207	221	151	153	80	82
ASWAD BALADI / ISR36-2013-5	**5**	177	183	259	259	150	150	245	247	263	271	171	187	232	234	191	199	246	250	245	253	203	207	151	153	80	82
BUSTAN / ISR36-2013-16	**16**	177	177	245	259	132	138	247	249	271	271	167	187	236	236	191	203	240	242	249	253	207	215	151	157	82	84
YAEL / ISR36-2013-21	**21**	173	177	m.d	m.d	138	150	247	249	255	271	167	187	m.d	m.d	191	203	240	249	249	255	207	217	151	157	78	84
BEER / ISR36-2013-46	**46**	177	183	259	259	138	154	249	253	249	249	167	187	234	234	199	203	242	246	245	253	207	215	151	153	82	84
ISR036-2013-98	**98**	185	191	217	237	130	140	239	247	253	271	167	183	228	228	191	199	240	240	249	255	207	211	151	153	78	78
ISR036-2013-15	**15**	177	183	249	259	140	150	247	247	261	271	171	187	235	235	191	203	246	250	245	245	203	207	153	153	80	82
ISR036-2013-61	**61**	183	191	237	259	140	150	239	249	241	251	167	189	232	234	187	191	240	256	249	255	207	207	151	157	78	80
ISR036-2013-64	**64**	177	191	245	259	150	152	233	239	255	273	183	187	226	236	187	195	246	250	247	255	211	221	151	153	78	84
ISR036-2013-67	**67**	179	183	m.d	m.d	138	152	243	251	m.d	m.d	167	187	m.d	m.d	187	203	246	254	247	247	207	207	151	151	80	84
ISR036-2013-69	**69**	179	183	259	259	148	152	243	249	259	271	189	189	232	246	187	199	246	250	249	249	207	221	151	151	78	80
ISR036-2013-74	**74**	179	183	257	257	150	152	243	251	255	271	167	187	238	240	189	203	248	262	249	249	207	207	151	151	80	84

SATIVA		SSR loci																		
**Accession name / code**	**Internal code**	**VVIH54**	**VVIH54**	**VVIV37**	**VVIV37**	**VMC4F8**	**VMC4F8**	**VVMD21**	**VVMD21**	**VVIN73**	**VVIN73**	**VMC4F3.1**	**VMC4F3.1**	**VVIB01**	**VVIB01**	**VVIP31**	**VVIP31**	**VVIV67**	**VVIV67**	**Synonymous**
BITUNI TORAN / ISR36-2013-9004	**9004**	166	178	160	162	113	121	249	265	266	266	165	189	291	295	179	187	368	370	
HADARI / ISR36-2013-9006	**9006**	168	178	158	180	117	119	249	255	266	270	179	203	295	295	189	191	356	356	
DUMIAT / ISR36-2013-9007	**9007**	166	178	158	180	111	115	243	265	266	268	183	187	291	295	179	179	350	356	Marawi gush hachalav
MAATAR / ISR36-2013-9008	**9008**	166	176	154	154	117	121	249	249	266	266	183	189	291	291	173	185	356	372	
SAFADI / ISR36-2013-9009	**9009**	178	180	152	160	117	123	249	255	266	266	189	203	291	291	179	191	356	372	Zeinisheik(Klein et al), Asba el arus
MARAWANI / ISR36-2013-9011	**9011**	160	168	158	158	117	121	243	249	266	266	183	187	291	291	173	177	356	358	Darwishi(Basheer et al)
BALUTI / ISR36-2013-9012	**9012**	166	166	152	180	111	123	243	255	266	266	183	203	291	295	179	189	350	356	Bituni (Klein et al), Bituni aruv
ZEITUNI / ISR36-2013-9013	**9013**	138	166	158	162	115	117	249	265	266	266	163	189	291	291	183	185	350	350	Karkashani
RUMI / ISR36-2013-9015	**9015**	166	176	166	166	113	117	243	249	266	266	187	201	291	291	183	185	372	388	
MIDWAR / ISR36-2013-9017	**9017**	166	178	166	166	117	123	249	265	266	268	187	189	291	307	185	187	358	358	Dorubeli
TOFACHI / ISR36-2013-9018	**9018**	166	166	158	160	123	123	243	249	266	266	189	203	291	295	177	191	372	388	Shami(Klein et al)
DABOUKI MASRIK / ISR36-2013-9019	**9019**	166	176	160	160	123	123	243	255	266	266	203	203	291	291	179	183	358	356	Dabuki (Eur. vitis database), Salati, Kasuti,, Zani baladi, Dabuki karniel
DARWISHI / ISR36-2013-9024	**9024**	166	166	166	166	123	123	243	249	266	266	187	189	291	291	185	187	358	358	
SIKUSI / ISR36-2013-9026	**9026**	166	166	158	160	123	123	249	265	266	266	189	203	295	295	177	183	356	372	
HILWANI / ISR36-2013-9028	**9028**	160	178	152	174	119	121	249	249	266	266	163	189	291	291	185	189	360	368	
TAMAR CHULATA / ISR36-2013-9029	**9029**	166	178	148	162	111	123	255	255	266	266	165	189	291	295	173	183	356	360	Dattier de beyrouth(european vitis database)
ZENI ARUV / ISR36-2013-9031	**9031**	166	178	160	160	119	121	249	255	266	266	183	187	291	295	183	187	358	358	
JANDALI / ISR36-2013-9034	**9034**	166	176	158	158	113	117	255	265	266	266	187	203	291	291	179	183	356	358	
MARAWI / ISR36-2013-9036	**9036**	166	176	152	160	117	123	249	265	266	266	203	203	291	291	183	191	356	356	Marawi taweel(Basheer et al), Karashi, Dabuki aruv
ASWAD BALADI / ISR36-2013-5	**5**	166	176	160	160	123	123	243	249	266	266	187	189	291	295	179	183	356	372	
BUSTAN / ISR36-2013-16	**16**	166	166	152	158	113	115	249	265	266	266	173	175	291	291	179	179	356	360	
YAEL / ISR36-2013-21	**21**	160	166	152	162	113	123	249	265	266	266	173	203	291	307	175	179	360	372	
BEER / ISR36-2013-46	**46**	138	166	160	176	117	125	255	265	266	266	187	187	291	291	179	183	356	388	
ISR036-2013-98	**98**	166	166	152	162	119	123	249	255	258	266	173	183	291	291	183	191	356	356	
ISR036-2013-15	**15**	166	176	152	160	117	123	243	255	266	266	187	203	291	295	179	183	350	356	
ISR036-2013-61	**61**	160	176	152	180	121	123	243	249	266	266	179	189	291	295	189	191	346	368	
ISR036-2013-64	**64**	138	160	152	160	115	121	249	265	266	266	203	203	291	295	179	189	356	358	
ISR036-2013-67	**67**	166	176	158	160	113	117	m.d	m.d	m.d	m.d	187	203	291	291	179	183	372	388	
ISR036-2013-69	**69**	176	176	m.d	m.d	117	123	243	255	m.d	m.d	173	181	291	295	189	191	358	372	
ISR036-2013-74	**74**	166	176	158	160	113	117	243	249	266	266	187	201	291	291	183	185	372	388	

Sativa		SSR loci																									
**Accession name / code**	**Internal code**	**VVMD27**	**VVMD27**	**VVMD28**	**VVMD28**	**VVS2**	**VVS2**	**VVMD7**	**VVMD7**	**VVMD32**	**VVMD32**	**VMC1B11**	**VMC1B11**	**VVMD5**	**VVMD5**	**VrZAG62**	**VrZAG62**	**VVMD25**	**VVMD25**	**VrZAG79**	**VrZAG79**	**VVMD24**	**VVMD24**	**VVIN16**	**VVIN16**	**VVIQ52**	**VVIQ52**
ISR036-2013-99	**99**	177	177	245	261	132	148	247	249	255	259	171	187	238	238	203	203	242	256	239	245	207	217	151	151	80	80
ISR036-2014-131	**131**	177	183	237	245	140	140	239	249	251	271	167	189	232	236	187	197	246	256	239	251	207	215	153	157	78	84
ISR036-2014-177	**177**	177	177	245	261	130	148	247	251	257	273	171	171	236	244	191	203	242	256	249	255	207	215	153	157	78	78
ISR036-2014-178	**178**	173	179	247	259	140	154	247	247	257	271	171	175	236	238	187	191	246	256	249	251	207	215	151	157	80	84
ISR036-2014-181	**181**	179	183	259	271	138	140	245	247	251	263	167	189	234	234	195	203	242	246	247	247	207	207	151	153	80	84
ISR036-2014-182	**182**	179	179	237	259	132	138	247	251	263	271	167	189	234	236	203	203	242	246	247	247	211	221	153	153	82	86
ISR036-2014-183	**183**	179	183	237	237	148	150	249	251	263	271	167	173	236	236	185	203	246	250	239	247	211	211	151	153	82	84
ISR036-2014-187	**187**	177	177	237	259	130	130	239	249	253	263	167	171	236	244	199	203	240	246	247	247	207	207	153	153	80	80
ISR036-2014-191	**191**	177	179	241	259	130	132	233	247	255	263	171	179	230	232	203	203	246	268	249	251	205	211	151	151	78	78
ISR036-2014-220	**220**	177	183	249	261	148	150	245	247	251	271	167	189	226	234	191	199	242	250	247	247	211	221	149	151	78	82
ISR036-2014-238	**238**	177	183	255	273	140	140	247	247	257	257	167	189	232	232	203	203	246	246	251	251	205	215	151	153	78	84
ISR036-2014-241	**241**	183	189	237	253	132	138	247	255	261	271	173	189	234	236	191	199	248	256	239	251	205	211	149	151	80	82
ISR036-2014-243	**243**	179	179	259	259	140	148	249	249	257	261	167	189	232	234	199	203	246	250	239	251	205	221	151	151	78	80
ISR036-2015-291	**291**	179	179	247	259	128	150	243	243	251	261	167	171	236	236	187	187	240	250	251	251	207	207	151	151	80	82
ISR036-2014-292	**292**	177	183	245	259	140	150	243	247	259	271	167	171	220	234	181	189	246	250	237	257	207	211	149	151	82	84
ISR036-2015-310	**310**	177	177	245	259	132	148	245	247	263	271	167	179	236	244	191	201	242	242	239	239	205	207	151	151	78	84
ISR036-2015-312	**312**	177	177	245	259	152	154	239	247	263	271	167	173	m.d	m.d	187	191	246	246	247	255	205	207	149	151	80	84
ISR036-2015-315	**315**	177	183	235	269	132	132	239	249	251	271	185	185	224	230	185	187	256	256	251	255	207	211	151	157	80	84
ISR036-2015-318	**318**	177	183	245	245	132	132	245	273	273	273	167	173	230	236	185	191	256	256	239	257	211	211	157	157	78	82
ISR036-2015-320	**320**	179	187	243	249	140	144	231	239	241	251	185	193	226	226	187	193	242	256	251	259	211	213	153	159	78	80
ISR036-2015-322	**322**	183	189	243	259	132	132	239	249	241	271	185	195	226	230	185	193	256	256	243	255	201	207	151	155	78	80
ISR036-2015-350	**350**	177	191	247	261	152	152	243	243	271	271	183	189	230	244	187	187	246	246	249	249	207	207	151	157	78	80
ISR036-2015-352	**352**	177	179	259	259	140	148	239	249	261	263	167	167	232	232	195	203	246	246	239	251	205	221	151	157	80	82
ISR036-2015-356	**356**	177	177	259	259	132	148	243	253	257	271	173	189	244	244	187	191	240	242	255	259	207	211	153	157	78	84
ISR036-2015-369	**369**	177	191	259	259	150	152	247	249	257	263	179	185	226	238	187	203	246	250	247	249	215	221	157	157	78	80
ISR036-2015-372	**372**	177	177	235	259	132	148	243	253	255	271	173	189	244	244	187	191	240	242	251	257	207	211	153	157	78	84
ISR036-2015-380	**380**	179	191	245	259	130	148	239	249	257	271	167	189	232	236	187	203	246	250	249	251	205	211	151	151	76	76
ISR036-2015-381	**381**	m.d	m.d	237	245	144	148	251	263	251	263	m.d	m.d	226	234	199	203	242	246	243	257	207	211	151	151	80	84
ISR036-2015-386	**386**	179	191	245	245	130	148	239	245	251	257	167	173	230	236	187	203	240	250	255	257	211	211	151	157	78	84
ISR036-2015-389	**389**	179	183	253	259	130	144	245	245	255	255	173	179	230	244	199	201	240	252	247	251	211	213	151	153	70	80
ISR036-2015-397	**397**	177	177	245	259	127	148	239	247	251	261	167	179	244	244	191	195	238	246	243	243	205	207	149	151	80	82

SATIVA		SSR loci																	
**Accession name / code**	**Internal code**	**VVIH54**	**VVIH54**	**VVIV37**	**VVIV37**	**VMC4F8**	**VMC4F8**	**VVMD21**	**VVMD21**	**VVIN73**	**VVIN73**	**VMC4F3.1**	**VMC4F3.1**	**VVIB01**	**VVIB01**	**VVIP31**	**VVIP31**	**VVIV67**	**VVIV67**
ISR036-2013-99	**99**	176	178	158	180	111	117	243	249	266	266	175	183	291	295	177	191	356	370
ISR036-2014-131	**131**	166	176	150	180	117	123	243	255	266	266	179	203	291	295	189	191	346	356
ISR036-2014-177	**177**	166	166	176	176	111	117	243	249	266	266	183	187	291	291	179	189	350	356
ISR036-2014-178	**178**	160	178	152	158	113	117	249	249	266	266	169	203	291	299	179	191	360	374
ISR036-2014-181	**181**	166	180	152	154	m.d	m.d	249	249	266	266	179	203	291	291	189	191	356	358
ISR036-2014-182	**182**	160	166	154	160	113	123	243	255	266	266	181	203	291	295	173	179	356	360
ISR036-2014-183	**183**	158	160	158	170	115	119	243	255	266	266	173	181	295	295	179	191	360	388
ISR036-2014-187	**187**	166	166	160	160	115	117	243	255	266	266	179	189	291	291	183	189	358	388
ISR036-2014-191	**191**	174	176	150	152	117	123	249	249	258	266	203	213	291	291	183	183	356	364
ISR036-2014-220	**220**	166	166	152	158	117	123	243	249	266	266	187	187	291	295	179	183	350	356
ISR036-2014-238	**238**	166	166	m.d	m.d	111	117	255	255	266	266	183	183	295	295	191	191	356	356
ISR036-2014-241	**241**	156	166	160	170	107	119	221	255	266	268	207	231	295	299	187	209	366	370
ISR036-2014-243	**243**	166	176	150	150	117	123	243	255	266	266	183	189	291	295	189	191	356	374
ISR036-2015-291	**291**	168	178	152	162	121	123	243	249	266	266	183	203	291	295	183	183	356	360
ISR036-2014-292	**292**	166	180	150	170	117	121	249	255	266	266	183	187	291	291	189	191	356	356
ISR036-2015-310	**310**	166	166	158	158	119	127	243	243	266	266	183	189	291	295	177	179	356	388
ISR036-2015-312	**312**	166	166	158	174	111	121	243	255	266	266	187	189	291	295	191	191	m.d	m.d
ISR036-2015-315	**315**	166	166	150	150	113	113	243	255	258	266	185	189	295	299	173	185	m.d	m.d
ISR036-2015-318	**318**	166	166	160	162	119	119	255	255	266	266	173	173	295	295	187	187	370	388
ISR036-2015-320	**320**	166	168	162	162	113	119	229	255	266	266	173	187	291	295	177	193	362	374
ISR036-2015-322	**322**	168	178	162	162	109	111	227	255	266	266	169	167	291	295	173	193	326	356
ISR036-2015-350	**350**	160	166	170	174	119	119	243	249	266	266	179	187	291	291	189	189	356	388
ISR036-2015-352	**352**	176	178	150	160	115	123	243	265	266	268	189	189	291	291	191	191	356	358
ISR036-2015-356	**356**	140	140	152	160	115	119	257	265	268	267	183	203	m.d	m.d	179	191	350	358
ISR036-2015-369	**369**	166	166	150	180	117	123	243	249	266	266	183	187	291	291	185	191	346	378
ISR036-2015-372	**372**	178	178	152	160	115	119	257	265	266	266	183	203	291	291	179	191	350	358
ISR036-2015-380	**380**	176	176	150	180	117	117	243	249	266	266	189	203	291	295	183	189	354	356
ISR036-2015-381	**381**	166	166	158	170	113	121	243	265	258	266	183	189	291	291	175	191	356	388
ISR036-2015-386	**386**	166	168	150	158	123	125	249	249	266	266	183	189	291	295	191	191	356	388
ISR036-2015-389	**389**	140	166	150	160	125	125	249	255	266	266	183	183	295	295	179	181	356	368
ISR036-2015-397	**397**	166	178	158	180	111	115	243	265	266	268	183	187	291	295	179	179	m.d	m.d

Sylvestris		SSR loci																									
Accession name / code	Internal code	VVMD27	VVMD27	VVMD28	VVMD28	VVS2	VVS2	VVMD7	VVMD7	VVMD32	VVMD32	VMC1B11	VMC1B11	VVMD5	VVMD5	VrZAG62	VrZAG62	VVMD25	VVMD25	VrZAG79	VrZAG79	VVMD24	VVMD24	VVIN16	VVIN16	VVIQ52	VVIQ52
ISR036-2013-1	**1**	179	183	245	249	132	140	245	249	255	259	179	185	232	244	199	199	240	242	245	245	211	215	153	153	80	84
ISR036-2013-2	**2**	177	177	259	271	130	138	239	245	251	255	173	189	230	234	199	201	242	252	245	247	203	211	151	153	84	84
ISR036-2013-3	**3**	177	177	259	259	130	134	247	249	249	257	187	187	234	246	199	203	240	242	245	249	207	211	149	153	80	84
ISR036-2013-4	**4**	177	177	245	245	132	144	245	249	253	257	171	179	232	236	193	201	252	256	247	247	211	221	153	153	80	86
ISR036-2013-22	**22**	177	177	249	255	140	148	249	249	245	255	183	183	226	234	193	193	240	240	245	245	215	217	151	153	78	80
ISR036-2013-23	**23**	177	177	245	249	132	150	249	249	243	271	171	183	m.d	m.d	193	203	242	246	245	247	215	217	153	153	78	80
ISR036-2013-24	**24**	177	177	249	255	132	148	247	249	255	255	173	183	m.d	m.d	193	203	240	250	242	245	203	217	153	153	78	80
ISR036-2013-48	**48**	177	183	255	255	132	140	239	249	245	255	163	183	226	236	193	201	240	242	245	249	211	211	153	153	78	80
ISR036-2013-50	**50**	177	177	255	255	148	150	247	249	253	271	173	183	232	236	193	203	240	246	245	247	211	217	153	153	78	78
ISR036-2013-51	**51**	177	177	255	255	142	150	247	249	253	259	171	183	226	234	193	203	240	246	241	245	211	217	153	153	78	80
ISR036-2013-54	**54**	179	179	245	269	138	140	247	249	255	261	171	183	232	234	199	203	240	252	245	247	203	211	151	151	72	72
ISR036-2013-56	**56**	177	179	269	281	130	138	245	249	253	261	171	173	230	246	199	201	242	252	245	249	203	211	151	153	72	78
ISR036-2013-59	**59**	179	179	245	269	126	130	245	245	249	253	179	187	m.d	m.d	193	201	240	240	245	247	211	211	153	153	82	88
ISR036-2014-128	**128**	177	183	245	245	140	150	247	249	243	243	171	185	234	244	199	203	240	248	247	251	215	215	151	153	78	84
ISR036-2014-141	**141**	177	179	245	249	132	146	233	251	251	271	167	185	222	244	203	203	240	256	247	257	211	217	151	153	80	82
ISR036-2014-142	**142**	m.d	m.d	m.d	m.d	142	150	233	247	255	271	173	189	232	244	203	203	240	240	245	247	211	211	151	153	72	82
ISR036-2014-143	**143**	177	179	245	259	132	134	249	253	257	275	173	179	232	236	187	195	240	256	243	251	207	209	149	151	80	84
ISR036-2014-144	**144**	179	183	249	249	140	142	249	259	251	251	171	179	230	236	195	201	240	242	247	247	211	221	151	153	78	84
ISR036-2014-145	**145**	177	177	245	259	132	150	233	245	257	271	171	179	222	244	193	195	240	250	247	255	211	213	151	153	78	80
ISR036-2014-146	**146**	177	179	245	259	132	142	239	245	251	261	179	195	232	234	m.d	m.d	240	242	237	247	205	207	151	153	78	80
ISR036-2014-147	**147**	177	177	245	259	130	132	247	247	251	255	179	185	234	244	203	203	240	256	247	251	205	211	153	153	80	80
ISR036-2014-148	**148**	177	177	245	245	130	132	239	247	261	261	171	185	234	236	195	203	240	242	245	247	207	215	149	151	78	82
ISR036-2014-149	**149**	177	179	237	249	130	132	247	249	261	271	171	179	234	234	193	203	240	240	245	245	215	217	149	153	78	84
ISR036-2014-150	**150**	177	183	237	245	132	132	247	247	m.d	m.d	185	185	236	236	191	195	240	240	247	255	205	211	151	153	78	80
ISR036-2014-151	**151**	177	183	237	245	132	132	247	249	257	271	175	179	234	236	191	193	240	240	245	255	211	217	151	153	78	80
ISR036-2014-152	**152**	177	191	259	259	132	134	245	247	257	271	171	189	234	236	195	203	244	252	245	249	211	217	153	153	78	80
ISR036-2014-153	**153**	177	191	241	253	132	142	249	259	251	255	179	189	244	244	193	195	240	240	247	249	211	221	151	153	80	84
ISR036-2014-156	**156**	177	191	253	259	134	140	233	239	257	271	167	175	236	236	195	203	244	252	245	247	211	211	153	153	80	84
ISR036-2014-157	**157**	177	191	245	259	134	140	239	247	257	263	171	179	236	244	195	203	242	252	245	249	211	211	153	153	78	84
ISR036-2014-158	**158**	177	183	249	253	132	142	233	239	257	271	167	171	222	236	189	195	240	256	247	255	211	221	151	153	80	80
ISR036-2014-159	**159**	173	177	245	261	134	148	247	249	257	271	167	189	234	236	189	195	242	244	247	255	207	211	151	151	84	84
ISR036-2014-160	**160**	177	179	245	253	132	134	245	247	257	271	171	189	236	236	195	203	244	252	245	249	211	217	153	153	78	84

Sylvestris		SSR loci																	
**Accession name / code**	**Internal code**	**VVIH54**	**VVIH54**	**VVIV37**	**VVIV37**	**VMC4F8**	**VMC4F8**	**VVMD21**	**VVMD21**	**VVIN73**	**VVIN73**	**VMC4F3.1**	**VMC4F3.1**	**VVIB01**	**VVIB01**	**VVIP31**	**VVIP31**	**VVIV67**	**VVIV67**
ISR036-2013-1	**1**	176	178	160	168	105	115	249	249	m.d	m.d	183	197	295	295	189	193	356	378
ISR036-2013-2	**2**	138	138	154	154	115	123	249	249	258	266	179	181	291	291	183	185	356	360
ISR036-2013-3	**3**	160	162	148	148	119	121	249	255	266	266	167	181	295	295	177	185	356	356
ISR036-2013-4	**4**	138	154	152	158	119	123	255	265	266	268	183	187	295	307	183	183	356	360
ISR036-2013-22	**22**	166	170	154	160	115	119	249	249	m.d	m.d	185	189	295	297	183	185	350	362
ISR036-2013-23	**23**	152	162	154	154	123	123	249	249	266	266	185	185	295	295	185	185	362	362
ISR036-2013-24	**24**	134	152	148	154	115	123	249	249	m.d	m.d	183	185	295	295	185	191	362	362
ISR036-2013-48	**48**	162	166	154	154	105	123	249	249	m.d	m.d	181	185	295	295	185	185	356	362
ISR036-2013-50	**50**	154	162	148	160	123	123	249	249	266	266	189	189	295	295	185	185	362	366
ISR036-2013-51	**51**	162	162	154	172	123	123	249	249	266	266	189	189	295	295	181	185	362	362
ISR036-2013-54	**54**	138	160	154	160	105	121	249	249	m.d	m.d	181	189	291	295	179	181	350	360
ISR036-2013-56	**56**	138	160	154	160	121	123	249	249	258	268	183	189	291	291	177	179	360	362
ISR036-2013-59	**59**	138	138	168	168	123	123	249	249	m.d	m.d	189	189	295	295	181	189	360	360
ISR036-2014-128	**128**	138	178	m.d	m.d	105	123	249	249	268	270	181	183	291	295	183	193	356	380
ISR036-2014-141	**141**	166	178	150	180	119	123	243	255	266	272	183	189	295	299	179	189	350	362
ISR036-2014-142	**142**	166	176	152	152	115	115	249	249	258	266	183	183	295	311	187	191	356	356
ISR036-2014-143	**143**	166	166	150	174	113	119	243	255	266	266	183	223	291	295	179	193	354	362
ISR036-2014-144	**144**	166	166	150	158	119	123	243	249	266	270	165	175	291	299	195	181	356	356
ISR036-2014-145	**145**	166	180	158	166	113	125	243	249	258	268	181	187	291	291	195	187	356	356
ISR036-2014-146	**146**	168	178	158	158	117	125	249	255	266	274	183	183	291	291	179	179	354	354
ISR036-2014-147	**147**	166	180	164	170	123	123	243	249	258	270	183	223	295	295	179	191	356	380
ISR036-2014-148	**148**	160	164	152	160	123	123	243	243	258	266	179	183	291	295	183	187	356	356
ISR036-2014-149	**149**	164	178	m.d	m.d	113	123	243	265	258	258	179	183	291	295	179	183	356	362
ISR036-2014-150	**150**	166	178	158	158	113	113	249	249	266	266	181	223	295	295	179	179	356	356
ISR036-2014-151	**151**	176	178	158	158	113	123	249	265	258	266	183	223	291	295	179	183	350	356
ISR036-2014-152	**152**	164	180	150	160	113	117	249	255	268	274	175	183	291	295	179	183	362	362
ISR036-2014-153	**153**	166	168	158	158	113	123	249	249	258	266	181	189	291	307	187	191	356	362
ISR036-2014-156	**156**	168	178	150	160	113	117	247	265	266	274	183	183	295	307	179	183	362	388
ISR036-2014-157	**157**	166	168	158	168	113	125	243	265	258	266	181	183	291	307	175	179	360	362
ISR036-2014-158	**158**	168	178	158	176	113	117	249	255	258	274	175	183	295	295	175	179	356	362
ISR036-2014-159	**159**	168	178	158	176	117	117	249	255	266	274	181	183	295	311	175	191	356	362
ISR036-2014-160	**160**	168	178	158	176	113	117	247	249	268	274	183	189	291	295	179	183	356	362

**Sylvestris**		**SSR loci**																											
**Accession name / code**	**Internal code**	**VVMD27**	**VVMD27**	**VVMD28**	**VVMD28**	**VVS2**	**VVS2**	**VVMD7**	**VVMD7**	**VVMD32**	**VVMD32**	**VMC1B11**	**VMC1B11**	**VVMD5**	**VVMD5**	**VrZAG62**	**VrZAG62**	**VVMD25**	**VVMD25**	**VrZAG79**	**VrZAG79**	**VVMD24**	**VVMD24**	**VVIN16**	**VVIN16**	**VVIQ52**	**VVIQ52**
ISR036-2014-162	**162**	175	177	245	245	132	132	247	247	251	251	179	179	234	236	191	203	242	256	247	251	205	205	149	153	80	80
ISR036-2014-163	**163**	999	999	259	261	132	142	243	253	245	251	171	175	230	236	187	195	242	242	255	257	207	215	149	149	82	82
ISR036-2014-164	**164**	177	179	249	249	132	134	247	249	257	263	171	179	232	244	195	201	242	242	245	247	209	221	151	153	72	80
ISR036-2014-165	**165**	177	183	245	261	142	142	239	249	243	259	189	189	222	224	191	199	240	240	255	257	205	207	151	157	72	82
ISR036-2014-175	**175**	179	183	249	259	140	144	233	247	261	271	171	173	234	236	191	203	240	242	247	247	205	209	151	153	80	82
ISR036-2014-180	**180**	177	177	237	259	130	152	247	247	263	271	165	189	238	244	187	203	246	246	247	251	207	221	153	153	78	80
ISR036-2014-184	**184**	177	179	245	259	132	134	233	247	257	271	167	179	230	232	189	203	242	242	247	247	207	217	151	153	78	82
ISR036-2014-186	**186**	177	177	241	259	132	134	239	253	999	999	171	173	234	236	197	203	242	268	247	251	207	211	151	153	78	80
ISR036-2014-189	**189**	179	183	257	259	134	140	239	249	253	263	167	171	230	234	189	203	242	256	255	255	207	211	151	153	78	80
ISR036-2014-192	**192**	177	183	259	259	132	132	249	249	259	271	171	171	230	234	191	203	242	268	247	249	207	211	151	153	80	82
ISR036-2014-193	**193**	177	183	249	259	142	150	233	247	255	263	171	171	236	244	189	203	240	256	247	251	207	215	151	153	80	80
ISR036-2014-221	**221**	179	183	245	255	138	142	233	247	255	255	171	185	226	234	191	191	246	252	247	249	207	215	151	151	80	80
ISR036-2014-222	**222**	177	177	245	261	130	132	247	247	255	263	171	179	234	236	193	195	242	242	247	247	205	213	151	153	78	80
ISR036-2014-224	**224**	177	179	245	273	132	140	247	249	251	257	179	181	234	236	193	199	242	242	247	251	211	215	149	153	80	84
ISR036-2014-225	**225**	179	183	245	249	142	150	249	249	251	255	171	173	236	232	199	201	240	242	251	251	207	221	153	153	80	82
ISR036-2014-226	**226**	179	179	255	259	130	142	239	247	255	255	171	171	226	232	191	203	240	242	255	255	207	215	153	153	80	82
ISR036-2014-227	**227**	177	177	245	273	130	154	247	249	255	259	171	187	226	244	193	199	242	246	247	251	221	221	151	153	78	84
ISR036-2014-234	**234**	177	183	245	273	130	148	235	239	999	999	179	181	234	234	193	201	240	242	999	999	205	207	151	153	78	82
ISR036-2014-237	**237**	177	183	999	999	132	150	247	247	251	255	179	185	232	234	191	199	240	242	247	247	207	215	151	153	78	84
ISR036-2014-258	**258**	179	183	245	245	132	140	249	249	249	253	179	185	236	244	192	199	240	242	247	247	215	221	151	153	78	80
ISR036-2014-259	**259**	179	183	245	245	134	140	245	249	253	257	179	189	232	244	195	199	240	240	247	247	215	215	151	153	80	80
ISR036-2014-260	**260**	177	183	245	249	132	140	249	249	249	253	179	185	232	236	193	199	240	242	247	249	215	215	151	153	78	84
ISR036-2014-261	**261**	179	179	249	263	126	130	249	249	253	253	181	189	236	244	193	193	240	242	247	249	207	221	151	151	72	80
ISR036-2014-262	**262**	177	177	999	999	126	126	999	999	251	253	167	171	234	244	195	203	240	240	247	247	207	211	151	153	80	80
ISR036-2014-263	**263**	177	179	239	245	130	130	239	249	261	261	185	185	226	226	195	199	242	246	239	249	211	211	153	153	78	82
ISR036-2014-265	**265**	177	183	245	261	130	130	233	249	253	253	171	185	234	236	193	203	242	242	247	251	207	211	151	151	80	80
ISR036-2014-266	**266**	177	183	259	259	134	148	247	249	249	249	181	181	226	234	191	193	240	242	247	249	211	221	151	153	82	84
ISR036-2014-268	**268**	177	179	245	253	144	148	247	249	257	259	171	179	232	244	191	199	242	252	247	247	205	213	151	151	78	80
ISR036-2014-269	**269**	177	179	255	273	126	130	247	247	259	261	171	189	234	244	999	999	240	252	245	251	207	207	151	151	82	84
ISR036-2014-272	**272**	183	191	249	273	132	140	239	247	243	249	171	185	232	234	195	195	240	256	247	251	211	211	151	151	78	82
ISR036-2014-276	**276**	177	177	241	259	134	134	243	247	253	271	173	189	234	234	999	999	242	252	255	255	211	215	151	151	78	82

Sylvestris		SSR loci																	
**Accession name / code**	**Internal code**	**VVIH54**	**VVIH54**	**VVIV37**	**VVIV37**	**VMC4F8**	**VMC4F8**	**VVMD21**	**VVMD21**	**VVIN73**	**VVIN73**	**VMC4F3.1**	**VMC4F3.1**	**VVIB01**	**VVIB01**	**VVIP31**	**VVIP31**	**VVIV67**	**VVIV67**
ISR036-2014-162	**162**	138	180	152	168	117	123	249	265	270	270	163	183	291	295	175	187	354	356
ISR036-2014-163	**163**	160	166	150	152	117	119	247	249	m.d	m.d	169	179	291	301	173	179	356	374
ISR036-2014-164	**164**	166	166	150	160	117	125	247	255	258	266	181	183	291	299	181	183	356	356
ISR036-2014-165	**165**	164	166	152	164	119	123	247	249	266	274	165	225	291	299	181	191	350	356
ISR036-2014-175	**175**	168	182	152	168	117	125	255	265	266	268	183	203	291	295	175	187	356	368
ISR036-2014-180	**180**	152	168	176	176	115	121	243	243	266	266	165	189	291	299	189	191	358	368
ISR036-2014-184	**184**	166	166	150	160	115	125	249	249	258	270	181	183	291	291	181	189	356	362
ISR036-2014-186	**186**	166	166	168	170	117	119	249	249	258	274	165	183	295	295	183	191	354	360
ISR036-2014-189	**189**	162	178	152	160	113	117	249	255	266	270	165	209	291	295	179	185	362	380
ISR036-2014-192	**192**	164	178	m.d	m.d	117	123	243	249	266	266	169	183	291	295	179	183	356	362
ISR036-2014-193	**193**	162	164	158	168	113	117	253	255	258	266	169	183	291	291	179	183	356	360
ISR036-2013-196	**196**	164	166	154	160	115	123	249	249	m.d	m.d	159	185	295	295	185	185	362	362
ISR036-2014-197	**197**	166	166	148	160	105	117	249	255	266	270	181	183	295	295	177	179	356	356
ISR036-2014-221	**221**	150	158	160	160	115	119	249	249	258	270	183	183	295	295	179	183	360	362
ISR036-2014-222	**222**	138	162	154	154	117	123	249	249	266	266	181	187	295	295	181	183	360	380
ISR036-2014-224	**224**	138	160	150	150	103	123	249	249	266	270	181	189	295	295	173	183	356	362
ISR036-2014-225	**225**	138	138	150	154	117	125	249	265	268	270	163	181	295	295	173	185	350	356
ISR036-2014-226	**226**	166	168	154	154	117	119	249	249	268	270	163	163	295	295	181	183	350	350
ISR036-2014-227	**227**	152	168	154	154	111	123	249	255	266	266	179	189	291	295	181	191	354	380
ISR036-2014-234	**234**	160	166	150	168	119	119	249	249	266	270	181	187	291	295	183	191	356	368
ISR036-2014-237	**237**	138	162	150	160	103	123	249	249	258	270	187	187	295	295	183	187	356	362
ISR036-2014-258	**258**	176	178	160	160	103	103	249	249	270	270	183	189	295	295	177	193	356	360
ISR036-2014-259	**259**	m.d	m.d	154	160	115	117	249	249	266	270	183	189	295	295	183	193	356	356
ISR036-2014-260	**260**	138	178	160	160	103	123	249	249	266	270	183	189	295	295	183	189	356	380
ISR036-2014-261	**261**	138	138	154	168	103	123	249	249	264	266	187	187	291	295	177	177	360	362
ISR036-2014-262	**262**	168	168	168	168	123	123	249	249	264	264	163	187	291	295	187	187	356	360
ISR036-2014-263	**263**	162	178	150	150	115	117	243	249	258	274	163	181	295	295	189	193	354	360
ISR036-2014-265	**265**	138	160	154	168	119	123	249	249	270	270	187	187	295	295	177	187	362	380
ISR036-2014-266	**266**	m.d	m.d	160	160	119	123	249	265	266	270	169	189	291	295	181	181	356	356
ISR036-2014-268	**268**	138	178	150	160	123	123	249	249	260	268	179	185	295	295	181	183	356	388
ISR036-2014-269	**269**	160	166	m.d	m.d	123	123	243	249	270	274	183	187	291	291	173	179	356	368
ISR036-2014-272	**272**	160	166	158	158	121	123	249	255	258	266	169	179	291	295	173	183	356	360
ISR036-2014-276	**276**	176	178	168	168	113	123	249	249	266	266	203	213	291	295	179	183	354	362

***m.d- missing data**

Table S2. Classification of some Israeli accessions into proleses.

Accession	OIV202	OIV220	OIV223	proles
	Bunch: length	Berry: length	Berry: shape	
16	long	medium	ovate	orientalis
19	medium	medium	obovate	orientalis
9002	short	short	ovate	orientalis
9003	medium	long	ovate	orientalis
9004	medium	long	ovate	orientalis
9005	medium	medium	elliptic	pontica
9006	short	short	elliptic	occidentalis
9007	short	short	roundish	occidentalis
9009	medium	long	obtuse-ovate	orientalis
9011	long	long	obovate	orientalis
9012	very long	long	obovate	orientalis
9013	long	long	obovate	orientalis
9015	long	medium	flat	pontica
9016	very long	medium	roundish	pontica
9017	medium	medium	elliptic	pontica
9021	long	long	ovate	orientalis
9024	long	short	elliptic	orientalis
9025	medium	long	ovate	orientalis
9026	medium	medium	elliptic	pontica
9028	very long	long	roundish	orientalis
9029	very long	long	ovate	orientalis
9031	long	very long	ovate	orientalis
9034	long	long	obtuse-ovate	orientalis

*Accessions with bunch and berry length (OIV 202 and OIV 220 descriptors) classified as long or very long, and with berry shape (OIV 223) classified as ovate, obtuse-ovate or obovate were classified into the *proles orientalis*. Accessions with smaller bunch and berries and with berries roundish or elliptic were classified into the *proles pontica* (those with relatively larger grapes) or *occidentalis*.

Table S3. Accessions from FEM, Georgia and Central Asia

Population	Accession number<br>DNA collection	Accession name
VV1	904	**Garganega**
VV1	1465	Pergolin
VV1	1415	Bombino bianco
VV1	800	**Trebbiano toscano**
VV1	1160	**Malvasia di Candia**
VV1	1214	**Marzemina Bianca**
VV1	968	Humagne
VV1	92	Alionza
VV1	539	Coll. Romanesco Tipico 04
VV1	222	Bianco d' Alessano
VV1	489	Coda di volpe bianca
VV1	66	**Albana**
VV1	1692	Romane
VV1	201	Bellone
VV1	644	Braghina
VV1	2329	Vertzami
VV1	748	Forcese d' Ascoli
VV1	205	Beregi rozsas
VV1	2192	V.silvestris Pezzato 1
VV1	3044	Kashita
VV1	3047	Kazas
VV1	3062	Sinambel
VV1	3063	Serina Ezeze e Jenkishta
VV1	3064	Prishta
VV1	3092	Minishtir
VV2	1424	**Parellada**
VV2	1912	Torralba
VV2	749	Forgiarin
VV2	973	Imperatrice
VV2	1433	Patagonia
VV2	1741	Rutilia
VV2	662	**Malvar**
VV2	60	Akiki

Population	Accession number DNA collection	Accession name
VV2	1747	**Xarello**
VV2	625	Damaschino
VV2	57	**Airen**
VV2	130	Armenia chi 13
VV2	689	**Zalema**
VV2	111	Araignan
VV2	1289	Morrastel
VV2	1121	**Macabeu**
VV2	116	Arbois rose
VV2	1604	**Calmeria**
VV2	1023	Khalili Piskakes
VV2	327	**Canner seedles**
VV2	247	Bobal
VV2	1267	Molinera
VV2	1268	**Mollar**
VV2	1207	**Manto Negro**
VV2	549	**Beba**
VV2	93	Allaren
VV2	2336	Vijiriego
VV2	733	**Flame seedless**
VV2	1011	Katta kourgan
VV2	1863	Sultanina nera
VV2	90	Alicante nero
VV2	331	Canocazo
VV2	245	Boal de Natura
VV2	110	Aragatzi
VV3	2551	**Schiava Grossa**
VV3	153	Ascot Citronelle
VV3	1326	Muscat de st. Vallier blanc
VV3	1333	Muscat Lierval
VV3	1330	**Muscat Hamburg**
VV3	1344	Muskat Banatski

Population	Accession number DNA collection	Accession name
VV3	1353	Muskat Yantarnyi
VV3	1328	Muscat Fleur d'Oranger
VV3	1352	Muskat Vostochnyi
VV3	1657	Razaki
VV3	1068	Kozma Palne muskotaly
VV3	1378	Nektar
VV3	1874	Szoloskertek kiralynoje muskotaly
VV3	629	Daranyi Ignac
VV3	2317	Verduzzo
VV3	1278	Montonico bianco
VV3	1156	Malvasia del Lazio
VV3	2426	**Muscat of Alexandria**
VV3	672	Early Muscat
VV3	554	**Muscat à petit grains blanc**
VV3	884	Giovanna Mathiasz
VV3	336	Cardinal
VV3	547	Conegliano 213
VV4	2444	Montagna
VV4	1231	**Petit Meslier**
VV4	741	**Folle Blanche**
VV4	1678	Rieslaner
VV4	1348	Muskat Diamant
VV4	1406	Optima
VV4	411	**Chardonnay**
VV4	2438	Zweigelt blau
VV4	171	**Auxerrios**
VV4	167	**Aubin blanc**
VV4	1318	Muscardin
VV4	1379	Neoplanta
VV4	850	Geisenheim 341-58
VV4	872	GF 33-9-141 52-11-027
VV4	873	GF 37-28-75 52-11-064

Population	**Accession number** DNA collection	Accession name
VV4	1228	**Merlot blanc**
VV4	791	**Gamay**
VV4	1226	**Melon**
VV4	155	**Tinturié**
VV4	115	**Orbois**
Iran	IRZA13	Yaghoti red
Iran	IRZA12	Laal
Iran	IRZA03b	Shiraz seya yaghoti
Iran	IRZA04	Sahebi
Iran	IRZA05	Abak
Iran	IRZA06	Sefid daneh
Iran	IRZA08	Chesm gave
Iran	IRZA10	Shanei o beidaneh
Iran	IRZA11	Shast aroos
Iran	IRZB01	Yaghoti white
wild	2152	V.v. sylvestris
wild	2181	V.v. sylvestris
wild	2257	V.v. sylvestris
wild	2183	V.v. sylvestris
wild	2182	V.v. sylvestris
wild	2187	V.v. sylvestris
wild	2185	V.v. sylvestris
wild	2163	V.v. sylvestris
wild	2165	V.v. sylvestris
wild	2162	V.v. sylvestris
wild	2145	V.v. sylvestris
wild	2219	V.v. sylvestris
wild	2144	V.v. sylvestris
wild	2129	V.v. sylvestris
wild	2071	V.v. sylvestris
wild	2169	V.v. sylvestris
wild	2264	V.v. sylvestris

Population	Accession number DNA collection	Accession name
wild	2261	V.v. sylvestris
wild	2252	V.v. sylvestris
wild	2204	V.v. sylvestris
wild	2194	V.v. sylvestris
wild	2214	V.v. sylvestris
wild	2119	V.v. sylvestris
wild	2122	V.v. sylvestris
wild	2178	V.v. sylvestris
wild	2275	V.v. sylvestris
wild	2134	V.v. sylvestris
wild	2117	V.v. sylvestris
wild	2244	V.v. sylvestris
wild	2143	V.v. sylvestris
wild	2236	V.v. sylvestris
wild	2127	V.v. sylvestris
wild	2138	V.v. sylvestris
wild	2198	V.v. sylvestris
wild	2084	V.v. sylvestris
wild	2235	V.v. sylvestris
wild	2150	V.v. sylvestris
wild	2200	V.v. sylvestris
wild	2268	V.v. sylvestris
wild	2216	V.v. sylvestris
wild	2190	V.v. sylvestris
wild	2213	V.v. sylvestris
wild	2231	V.v. sylvestris
wild	2233	V.v. sylvestris
wild	2171	V.v. sylvestris
wild	2172	V.v. sylvestris
wild	2132	V.v. sylvestris

Population	Accession number<br>DNA collection	Accession name
sativa Iran	1161	Yaghoti red
sativa Iran	1162	Laal
sativa Iran	1166	Shiraz seya yaghoti
sativa Iran	1167	Sahebi
sativa Iran	1168	Abak
sativa Iran	1169	Sefid daneh
sativa Iran	1170	Chesm gave
sativa Iran	1171	Shanei o beidaneh
sativa Iran	1172	Shast aroos
sativa Iran	1173	Yaghoti white
sativa Central Asia	1001	Gavgur
sativa Central Asia	1002	Safali
sativa Central Asia	1005	Dzhavak
sativa Central Asia	1006	no name
sativa Central Asia	1007	Kishmish safed okruglii
sativa Central Asia	1008	Siekhi angur
sativa Central Asia	1009	Kishmish tajikskii
sativa Central Asia	1010	Surkhak white
sativa Central Asia	1011	Liali surkh
sativa Central Asia	1012	Shukurti surkh
sativa Central Asia	1013	Rozovii (Pink)
sativa Central Asia	1014	Siiokhi kalon umaraki
sativa Central Asia	1015	Bur angur
sativa Central Asia	1016	Chochi shtur doroz
sativa Central Asia	1018	Bishti
sativa Central Asia	1019	no name
sativa Central Asia	1020	Angur eie khonarotosh
sativa Central Asia	1021	Liali Yakdona
sativa Central Asia	1022	Mynosa
sativa Central Asia	1023	Liali khusha doroz
sativa Central Asia	1024	Obak surkhi
sativa Central Asia	1025	Siekh

Population	Accession number DNA collection	Accession name
sativa Central Asia	1026	Dzhaus safed turkani
sativa Central Asia	1027	Khusha doroz safed
sativa Central Asia	1028	Siiokhi maida kivrani
sativa Central Asia	1029	Eshvoi
sativa Central Asia	1030	Angur eie
sativa Central Asia	1031	Kishmish black tagansk
sativa Central Asia	1032	Angur surkhak
sativa Central Asia	1033	Sachal kara kashkadarinskiyi
sativa Central Asia	1034	Ak Lunda
sativa Central Asia	1037	Kok uzium
sativa Central Asia	1038	Mevagi khonatorosh
sativa Central Asia	1039	Doroi black tagansk
sativa Central Asia	1040	Bakhtiori black
sativa Central Asia	1043	Asma shaartuzsk
sativa Central Asia	1044	Vasarga black
sativa Central Asia	1045	Kara uzium
sativa Central Asia	1046	Sattar kara
sativa Central Asia	1047	Ichkimar white
sativa Central Asia	1048	Surkhak angur
sativa Central Asia	1049	Kizyl eshon uzium
sativa Central Asia	1051	Kishmish irtyshar
sativa Central Asia	1052	Ruzbari
sativa Central Asia	1053	Chini taifi
sativa Central Asia	1054	Kishmish krasnii rannii (red early)
sativa Central Asia	1055	Shurtak
sativa Central Asia	1056	Shtur angur
sativa Central Asia	1057	Rakhimi sufigi
sativa Central Asia	1058	Arom zobi
sativa Central Asia	1059	Mirza korak
sativa Central Asia	1061	Tukhumi kaftar
sativa Central Asia	1062	Mevagi
sativa Central Asia	1063	Kara gudung
sativa Central Asia	1064	Turdy govak

Population	Accession number<br>DNA collection	Accession name
sativa Central Asia	1065	Liali siekh
sativa Central Asia	1068	Vatan
sativa Central Asia	1069	Siekh rezak hermaphrodite
sativa Central Asia	1070	Choch vakh
sativa Central Asia	1071	Kuri boki
sativa Central Asia	1072	Ak uzium tagapskii
sativa Central Asia	1073	Irtyk apkar
sativa Central Asia	1074	Kara gushty
sativa Central Asia	1075	Sary uzium
sativa Central Asia	1157	Liuftaki kivrani
sativa Central Asia	1159	Chochi shutur siiokh
wild Central Asia	1076	V.v. sylvestris
wild Central Asia	1077	V.v. sylvestris
wild Central Asia	1079	V.v. sylvestris
wild Central Asia	1080	V.v. sylvestris
wild Central Asia	1082	V.v. sylvestris
wild Central Asia	1086	V.v. sylvestris
wild Central Asia	1087	V.v. sylvestris
wild Central Asia	1088	V.v. sylvestris
wild Central Asia	1089	V.v. sylvestris
wild Central Asia	1091	V.v. sylvestris
wild Central Asia	1092	V.v. sylvestris
wild Central Asia	1095	V.v. sylvestris
wild Central Asia	1096	V.v. sylvestris
sativa Georgia	1097	Tsnoris Tetra
sativa Georgia	1098	Chitistvala Bodburi
sativa Georgia	1099	Ubakluri
sativa Georgia	1100	Ikaltos Tsiteli
sativa Georgia	1101	Buera
sativa Georgia	1102	Kurkena
sativa Georgia	1103	Kisi
sativa Georgia	1104	Rkatsiteli Vardisperi
sativa Georgia	1105	Mtsvane Kakhuri

**Population**	**Accession number** DNA collection	**Accession name**
sativa Georgia	1108	Rkatsiteli Tsiteli
sativa Georgia	1109	Khikhvi Clone 430
sativa Georgia	1113	Chvitiluri
sativa Georgia	1114	Dziragoulis Shavi
sativa Georgia	1115	Shavstistska
sativa Georgia	1116	Tavtsitela
sativa Georgia	1117	Saperavi Budeshuriseburi
sativa Georgia	1119	Saperavi Clone 359
sativa Georgia	1122	Sapena
sativa Georgia	1124	Ghrubela Kakhuri
sativa Georgia	1129	Kharistvala Shavi
sativa Georgia	1130	Muradouli
sativa Georgia	1131	Ktsia
sativa Georgia	1135	Danakharuli
sativa Georgia	1136	Kikhuri
sativa Georgia	1137	Buza
sativa Georgia	1138	Didmtevana
sativa Georgia	1141	Krakhuba Clone
sativa Georgia	1142	Tsitska
sativa Georgia	1143	Bakhva
sativa Georgia	1144	Tsolikouri
sativa Georgia	1145	Berouli
sativa Georgia	1146	Melikuda Kartlis
sativa Georgia	1147	Jvari
sativa Georgia	1148	Vazisubnis Tsiteli
sativa Georgia	1149	Ghvinis Tsiteli
sativa Georgia	1151	Che-Khardani
sativa Georgia	1152	Argvetuli Sapere
sativa Georgia	1153	Dziganidzis Shavi
sativa Georgia	1155	Tavtsitela
sativa Georgia	1156	Bazaleturi

***BOLD = varieties true-to-type**
